# Catalytically Active Oxidized PtOx Species on SnO_2_ Supports Synthesized via Anion Exchange Reaction for 4-Nitrophenol Reduction

**DOI:** 10.3390/nano15151159

**Published:** 2025-07-28

**Authors:** Izabela Ðurasović, Robert Peter, Goran Dražić, Fabio Faraguna, Rafael Anelić, Marijan Marciuš, Tanja Jurkin, Vlasta Mohaček Grošev, Maria Gracheva, Zoltán Klencsár, Mile Ivanda, Goran Štefanić, Marijan Gotić

**Affiliations:** 1 Laboratory for Molecular Physics and Synthesis of New Materials, Division of Materials Physics, Ruđer Bošković Institute, Bijenička c. 54, 10000 Zagreb, Croatia; idjuras@irb.hr (I.Ð.); mohacek@irb.hr (V.M.G.); ivanda@irb.hr (M.I.); 2 Department of Physics, University of Rijeka, Radmile Matejčić 2, 51000 Rijeka, Croatia; rpeter@phy.uniri.hr; 3 National Institute of Chemistry, Hajdrihova 19, SI-1001 Ljubljana, Slovenia; goran.drazic@ki.si; 4 Petroleum and Petrochemical Department, Faculty of Chemical Engineering and Technology, University of Zagreb, Trg Marka Marulića Trg 19, 10000 Zagreb, Croatia; ffaragun@fkit.unizg.hr (F.F.); ranelic@fkit.unizg.hr (R.A.); 5 Division of Materials Chemistry, Ruđer Bošković Institute, Bijenička c. 54, 10000 Zagreb, Croatia; marijan.marcius@irb.hr; 6 Radiation Chemistry and Dosimetry Laboratory, Division of Materials Chemistry, Ruđer Bošković Institute, Bijenička c. 54, 10000 Zagreb, Croatia; tjurkin@irb.hr; 7 Budapest Neutron Centre, HUN-REN Centre for Energy Research, Konkoly-Thege Miklós út 29-33, 1121 Budapest, Hungary; maria.gracheva@ek.hun-ren.hu (M.G.); klencsar.zoltan@ek.hun-ren.hu (Z.K.)

**Keywords:** platinum, SnO_2_, anion exchange, Dowex 550, catalyst, 4-nitrophenol, XPS, ^119^Sn Mössbauer

## Abstract

An anion exchange-assisted technique was used for the synthesis of platinum-decorated SnO_2_ supports, providing nanocatalysts with enhanced activity for the reduction of 4-nitrophenol (4-NP) to 4-aminophenol (4-AP). In this study, a series of SnO_2_ supports, namely SnA (synthesized almost at room temperature), SnB (hydrothermally treated at 180 °C), and SnC (annealed at 600 °C), are systematically investigated, all loaded with 1 mol% Pt from H_2_PtCl_6_ under identical mild conditions. The chloride ions from the SnCl_4_ precursors were efficiently removed via a strong-base anion exchange reaction, resulting in highly dispersed, crystalline ~5 nm cassiterite SnO_2_ particles. All Pt/SnO_2_ composites displayed mesoporous structures with type IVa isotherms and H_2_-type hysteresis, with SP1a (Pt on SnA) exhibiting the largest surface area (122.6 m^2^/g) and the smallest pores (~3.5 nm). STEM-HAADF imaging revealed well-dispersed PtOx domains (~0.85 nm), while XPS confirmed the dominant Pt^4+^ and Pt^2+^ species, with ~25% Pt^0^ likely resulting from photoreduction and/or interactions with Sn–OH surface groups. Raman spectroscopy revealed three new bands (260–360 cm^−1^) that were clearly visible in the sample with 10 mol% Pt and were due to the vibrational modes of the PtOx species and Pt-Cl bonds introduced due the addition and hydrolysis of H_2_PtCl_6_ precursor. TGA/DSC analysis revealed the highest mass loss for SP1a (~7.3%), confirming the strong hydration of the PtOx domains. Despite the predominance of oxidized PtOx species, SP1a exhibited the highest catalytic activity (*k*_app_ = 1.27 × 10^−2^ s^−1^) and retained 84.5% activity for the reduction of 4-NP to 4-AP after 10 cycles. This chloride-free low-temperature synthesis route offers a promising and generalizable strategy for the preparation of noble metal-based nanocatalysts on oxide supports with high catalytic activity and reusability.

## 1. Introduction

Platinum-based catalysts have long been at the forefront of heterogeneous catalysis due to their exceptional activity, selectivity, and stability in a wide range of chemical reactions, including hydrogenation, oxidation, and environmental remediation processes [1]. In particular, platinum nanoparticles (PtNPs) on metal oxides provide a versatile platform for catalytic applications, as the nanoscale dispersion of Pt enhances the availability of active sites, while the support can influence the electronic properties and stability [2,3]. In addition to metallic PtNPs, recent studies have also investigated ionic platinum species as active catalytic centers, particularly in systems in which Pt is predominantly present in ionic states (e.g., Pt^2+^ or Pt^4+^). Okumura et al. [4] synthesized platinum catalysts supported on metal oxides, in particular CeO_2_, which contain highly dispersed ionic Pt species, mainly Pt^4+^ and Pt^2+^, and play a key role in catalytic performance. CeO_2_ was effective in stabilizing Pt^4+^, with some catalysts retaining over 70% Pt^4+^ even after repeated use. These ionic species were catalytically active and could be reversibly reduced to Pt^0^ under reaction conditions, forming dynamic active sites. The strong metal–support interaction and high dispersion of Pt^4+^ were crucial for high activity, stability and reusability in hydrosilylation reactions. Mukri et al. [5] presented the use of Pt-doped ionic catalysts in which Pt^2+^ contributes significantly to the catalytic activity of titanium(IV) oxide (TiO_2_). These ions, in conjunction with weakly bonded lattice oxygen, promote electron transfer and oxygen activation, which are essential for catalytic oxidation reactions. Pt^4+^ was significantly less active, as these ions occupied octahedral sites and, thus, lacked the structural and electronic effects necessary for catalysis. These ionic Pt catalysts have demonstrated unique reactivity and stability, opening new avenues for catalyst design. Barsan et al. [6] demonstrated that platinum can also to be present in its oxidized form. The oxidized platinum species, primarily as Pt^4+^—in PtO_2_-like clusters—are highly active and stable on the surface of SnO_2_. These species are not reduced under operating conditions and remain catalytically accessible. Their strong interaction with the SnO_2_ surface, probably via Pt–O–Sn linkages, enhances electron transfer and gas activation. Unlike metallic Pt, these oxidized Pt forms retain their activity even in humid environments, making them effective and durable catalytic centers for CO sensing in the presence of water vapor.

Bimetallic Pt-based catalysts offer enhanced catalytic activity, selectivity, and stability compared to pure Pt, while reducing the platinum content. Lai et al. [7] have reported on carefully engineered nanostructures, such as Pt-Pd or Pt-Ni alloys in core–shell or dendritic form, which show excellent performance in reactions, such as fuel cell operation and hydrogen evolution. A major drawback remains the difficulty in scaling up reproducible, controllable synthesis methods on a larger scale, which limits their wider application. Xu et al. [8] synthesized Pt-based ultrafine one-dimensional nanowires with superior catalytic activity and structural stability, making them suitable for advanced energy applications. Their elongated shape enhances transport properties and active site exposure, but challenges remain in scalable synthesis and precise control of their structure and composition, especially when scalable and reproducible methods are required. Recent advancements in Pt-based catalysts supported by carbon and conductive polymers have shown promise for direct methanol fuel cell anodes, especially with novel carbon materials, like nanotubes and graphene. Ramli and Kamarudin [9] report that alloying Pt with metals, like Fe, Co, and Ni, and designing advanced nanostructures, such as nanowires or hollow particles, significantly improves catalytic activity and stability while reducing Pt usage. However, challenges remain in scalable synthesis, high metal loading, and uniform dispersion on various carbon supports, particularly with high surface area materials. Zhan et al. [10] investigated non-carbon supports, such as metal oxides. Understanding and optimizing the strong metal–support interaction can significantly improve ORR performance. Single-atom catalysts offer high Pt utilization and catalytic efficiency, but future efforts need to focus on improving their stability and scalability for commercial applications. Among the various oxide supports, tin(IV) oxide (SnO_2_) has received increasing attention due to its high thermal stability, chemical stability, and ability to synergistically interact with Pt species, which can improve catalytic performance through strong metal–support interactions. Specifically, SnO_2_ offers the additional advantage of facilitating electron transfer and creating oxygen vacancies, which can enhance catalytic activity [11]. When used as a support for Pt nanoparticles, SnO_2_ has been shown to improve catalyst dispersion and stability, potentially leading to enhanced catalytic performance in redox reactions [12]. The catalytic efficiency of Pt/SnO_2_ systems is strongly influenced by the synthesis method and the purity of the final catalyst.

Building on our previous work, where we developed a microwave-assisted synthesis of Pt/SnO_2_ catalysts with high activity and reusability [13], we now take this a step further by improving this green synthesis approach. Specifically, we have developed a near room-temperature method for synthesizing both the SnO_2_ support and dispersing platinum, virtually without energy consumption. This advancement was made possible by removing chloride ions from the precursor solutions. By eliminating chloride during synthesis, we were able to obtain well-crystallized cassiterite-phase SnO_2_ at room temperature. Chloride removal is particularly important, as it is believed that chloride ions can hinder catalyst performance by poisoning active sites, disrupting nanoparticle dispersion, or altering the surface chemistry of the support [14]. This issue is especially pronounced in surface-sensitive reactions [15] or in aqueous environments where chloride can leach or promote undesirable side reactions [16]. To investigate how support properties influence platinum dispersion and catalytic behavior, we synthesized three distinct chloride-free SnO_2_ supports, as follows:

**SnA**, obtained via room-temperature precipitation following ion exchange;

**SnB**, produced by hydrothermal treatment of SnA at 180 °C, resulting in larger SnO_2_ particles;

**SnC**, generated by annealing SnB at 600 °C to further increase crystallinity and particle size.

These supports were then used to prepare Pt/SnO_2_ catalysts with identical platinum content. Notably, the platinum precursor (H_2_PtCl_6_) was dispersed at a temperature of only 40 °C, in contrast to the conventional methods that require temperatures around 300–400 °C. We were aware that such a low-temperature treatment would not result in the formation of metallic platinum nanoparticles. This assumption was confirmed by XPS analysis, which revealed the presence of oxidized Pt species (Pt^2+^ and Pt^4+^) on the surface of the SnO_2_ supports, forming PtOx/SnO_2_ catalysts. The catalytic performance of these PtOx/SnO_2_ catalysts was evaluated in the reduction of 4-nitrophenol (4-NP) to 4-aminophenol (4-AP). The results were correlated with the structural and textural properties of the catalysts, such as surface area and pore size distribution. By comparing materials that differ only in SnO_2_ particle size and crystallinity, this study aims to clarify the influence of support structure on platinum dispersion and overall catalytic efficiency.

## 2. Materials and Methods

### 2.1. Chemicals

Tin(IV) tetrachloride (Product No. 244678), manufactured by Sigma Aldrich (Steinheim, Germany), and hexachloroplatinic acid (CAS 18497-13-7), manufactured by ThermoFischer (Karlsruhe, Germany), were used as received. AmberLite HPR 550 ion exchange resin is supplied by Sigma Aldrich (St. Louis, MO, USA) in the form of translucent orange spheres with a particle size of 590 ± 50 μm. It was used after being rehydrated in deionized Milli-Q (MQ) water. The chemicals used for the catalytic experiments were as follows: 4-nitrophenol—Sigma Aldrich (Steinheim, Germany), Reagent Plus, ≥99%, CAS: 100-02-7, Product No.: 241.326 and sodium borohydride (NaBH_4_)—Alfa Aesar (Karlsruhe, Germany), min.98%, CAS: 16940-66-2, Product No.: 88983. Deionized Milli-Q water was used for catalytic experiments.

### 2.2. Stock Solution Preparation

For the SnCl_4_ stock solution, 35.06 g of the SnCl_4_·5 H_2_O powder was weighed out and dissolved in 50 mL of deionized Milli-Q water. For the H_2_PtCl_6_ stock solution, 5 g of the solid H_2_PtCl_6_·6 H_2_O was mixed with 4.93 mL of deionized Milli-Q water. The calculated concentrations of the tin and the platinum stock solutions were 2.0 mol dm^–3^ each (2M SnCl_4_ and 2M H_2_PtCl_6_).

### 2.3. Anion Exchange Resin Preparation

The anion exchange resin, which is supplied in dehydrated form, must be rehydrated to restore its functionality. This is achieved by immersing 160 g of resin in deionized MQ water, so that the volume of the resin submerged in MQ water is 400 mL (performed in a plastic 500 mL beaker). After 20 min of stirring (with a magnetic stirrer) to allow the resin beads to swell and fully hydrate, the resin is treated with a sodium hydroxide solution. In this step, the resin is converted to its hydroxide form, which increases its anion exchange capacity. The process involves mixing the resin with a 2M NaOH solution, rinsing the resin with MQ water and repeating the treatment with 2M NaOH. The resin is then thoroughly rinsed with MQ water to remove all residual NaOH and displaced Na^+^ and OH^–^ ions. This step is repeated until the rinse water reaches a neutral pH, indicating that no excess NaOH is present and confirming that the resin is in its desired hydroxide form.

The anion exchange resin used to remove chloride anions from the solution becomes saturated with chloride ions over time, so regeneration is required to restore its capacity for effective ion exchange. Regeneration converts the resin from the chloride form back to the hydroxide form, reactivating its anion exchange function. First, the regenerating NaOH solution must be prepared at a concentration of 6 to 8% by weight. The regenerating solution is then stirred with the resin for 15 min and then thoroughly rinsed twice with MQ water in a sieve to remove all residual NaOH and displaced chloride ions [17]. This process is repeated five or more times, depending on the condition of the anion exchange resin, until all chloride ions are removed from the resin beads, which is checked using silver nitrate (AgNO_3_) to exclude the presence of chloride ions. Once there is no more reaction, the resin is ready for reuse.

### 2.4. Synthesis of the Supports and Samples

**Figure 1:**
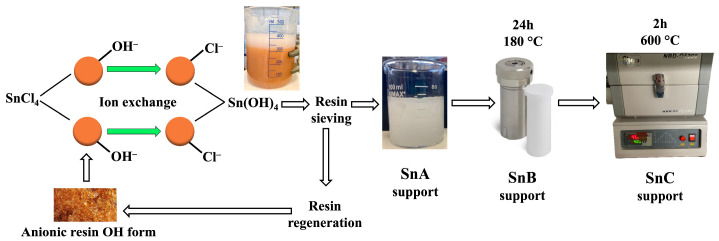
Anion exchange synthesis of SnA, SnB, and SnC supports.

For all three types of support, i.e., SnA, SnB, and SnC, 18 mL of 2 M SnCl_4_ was diluted with 102 mL of MQ water to prepare a 0.3 M SnCl_4_ solution. This solution was stirred for 20 min with a preconditioned anion exchange resin (the aforementioned 160 g of resin) to facilitate the removal of chloride ions by replacing them with hydroxide ions [17]. The resulting milky white suspension appeared after mixing for 20 min and reaching pH 3.5. It was carefully decanted, taking care not to entrain any resin beads. The entire support synthesis process is shown in Figure 1, while the precise steps are given in the next three paragraphs.

**Figure 2:**
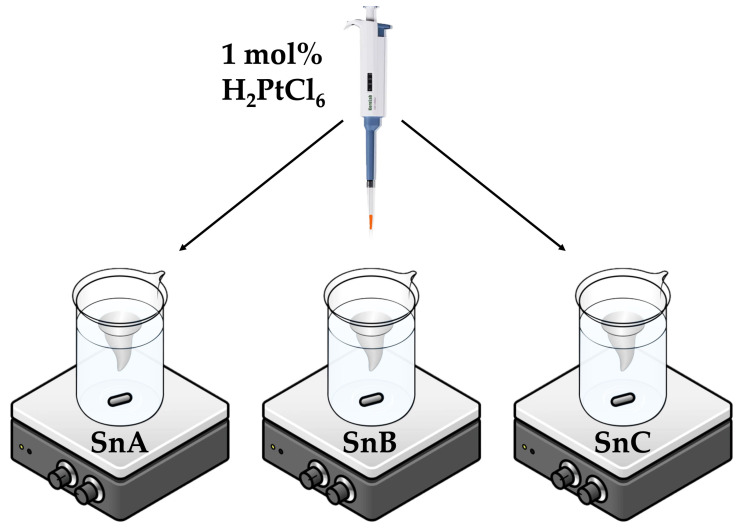
Platinum (H_2_PtCl_6_) was added to the three distinct SnO_2_ supports (SnA, SnB, and SnC) in the same way and in an identical amount—1 mol% Pt relative to the amount of SnO_2_ support.

For the SnA support, the suspension was quantitatively transferred to a glass beaker and stirred with a magnetic stirrer at 140 rpm and 40 °C for 72 h until complete evaporation of the water. The precipitate was dried overnight at 60 °C and then scraped from the beaker and homogenized using a mortar and pestle. Then, 1 g of the resulting white powder was stirred in 20 mL of MQ water with 35 µL of 2M H_2_PtCl_6_ to obtain a Pt loading of 1 mol% (Figure 2, identical for all three supports). This Pt-loaded SP1a sample was stirred with a magnetic stirrer at 140 rpm and 40 °C for 48 h, and then dried overnight at 60 °C. In the same manner, a sample with 10 mol% Pt was synthesized by adding 221 µL of 2M H_2_PtCl_6_ to 0.6 g of SnA support to obtain SP10a.

For the SnB support, the suspension was subjected to hydrothermal treatment in an autoclave at 180 °C for 24 h. After autoclaving, the suspension was transferred to a glass beaker with a magnetic stirrer and stirred at 140 rpm at 40 °C for 72 h to evaporate the water. The resulting precipitate (1 g) was dried overnight at 60 °C, collected, homogenized in a mortar and pestle, and used for platinum precipitation with 35 µL of 2M H_2_PtCl_6_ to reach a final Pt content of 1 mol%. The SP1b sample was stirred and dried, like the SP1a sample.

For the SnC support, the suspension was hydrothermally treated in an autoclave at 180 °C for 24 h. After autoclaving, the suspension was transferred to a glass beaker with a magnetic stirrer and stirred at 140 rpm at 40 °C for 72 h to evaporate the water. The powder was dried overnight at 60 °C and then annealed in a tube furnace at 600 °C for 2 h to enhance crystallinity. The annealed SnO_2_ (1 g) was then combined with 35 µL of 2M H_2_PtCl_6_ to achieve a Pt loading of 1 mol%. This SP1c sample was stirred and dried, like the SP1a and SP1b samples.

A table with the names of supports and samples, along with the synthesis condition summary, is given in the Supplementary Materials (Table S1).

### 2.5. Instrumental Analysis

X-ray diffraction (XRD) measurements were carried out at room temperature using an APD 2000 diffractometer (CuKα radiation, graphite monochromator, NaI-Tl detector) from ITALSTRUCTURES, Riva Del Garda, Italy.

Scanning electron microscopy (SEM) was performed with a Jeol Ltd. (Tokyo, Japan) 700F field emission SEM coupled to the EDS/INCA 350 system for energy-dispersive X-ray spectrometry (EDXS), constructed by Oxford Instruments Ltd. (Abingdon, UK).

An atomic resolution scanning transmission electron microscope (AR STEM), namely the Jeol ARM 200 CF model (JEOL Ltd., Tokyo, Japan) operating at 200 kV, was used for this study. This instrument was coupled to the Gatan Quantum ER system, which includes electron energy loss spectroscopy and energy-dispersive X-ray spectrometry capabilities using the Jeol Centurio 100 module.

For the nitrogen adsorption analysis performed at 77 K, the Quantachrome Autosorb iQ3 system (from Quantachrome Instruments, Boynton Beach, FL, USA) was used, which utilizes the Brunauer–Emmett–Teller (BET) technique to evaluate material properties. Prior to testing, a controlled heating process up to 250 °C was carried out under vacuum conditions to remove residual gases and moisture. The evacuation process was continued until the pressure fluctuations stopped increasing rapidly and reached a value below 50 millitorr per minute. Isothermal adsorption and desorption measurements were then performed at 77 K over a relative pressure range of about 10–5 up to almost 0.99.

X-ray photoelectron spectroscopy (XPS) was used to investigate the oxidation state of Sn and Pt in the oxidized Pt species on SnO_2_ supports. The analysis was carried out under ultra-high vacuum (UHV) conditions using a SPECS instrument (SPECS Surface Nano Analysis GmbH, Berlin, Germany). The experimental setup used an excitation energy of 1486.74 eV derived from the Al Kα X-ray emission and the Phoibos100 electron energy analyzer. To neutralize the charge accumulation in non-conductive oxide samples, a 5 eV electron flooding method was applied during the XPS analysis. A pass energy of 50 eV was chosen for the evaluation of the Pt 4f core levels, while a pass energy of 10 eV was used for the spectra around the Sn 3d levels. The experimental curves were fitted using a combination of Gaussian and Lorentz functions via the Unifit software 2024 (R. R. Hesse–UNIFIT Software, Leipzig, Germany) [18]. All photoemission spectra were calibrated using the C 1s peak, which was set at a binding energy (BE) of 284.5 eV.

Thermogravimetric analysis (TGA) was performed with a Mettler Toledo TGA/DSC 3+ instrument (Mettler Toledo, Schwerzenbach, Switzerland). In a typical experiment, 10-12 mg of the SnO_2_ support was placed in an aluminum oxide crucible and inserted into a furnace. The supports experienced non-isothermal heating from 35 °C to 1000 °C at a heating rate of 10 °C/min and a constant nitrogen (N_2_) gas flow at a rate of 50 mL/min. The thermogravimetric analysis and differential scanning calorimetry (DSC) data were recorded using a computer synchronized with the furnace. The differential thermogravimetry (DTG) data, generated from the first derivative of the TG, depicted the mass loss rate of the materials with increasing time or temperature. The thermal behavior and characteristic parameters of the supports in question were concluded from the TG and DTG data.

Then, ^119^Sn Mössbauer spectroscopy measurements were performed with a WissEl Mössbauer spectrometer (WissEl GmbH, Starnberg, Germany) setup operated in transmission geometry, with the samples and the source being kept at room temperature. A ^119m^Sn(CaSnO_3_) radioactive source (RITVERC JSC) with an activity of ~0.35 mCi provided the γ-rays. The source was driven by a sinusoidal velocity signal, with velocity extrema of approximately ±6 mm s^−1^. The raw spectra consisted of 2048 channels, which were subsequently folded into 1024 channels for further processing. The ^119^Sn isomer shift (*δ*) values are given relative to that of a SnO_2_ reference powder (Merck, Budapest, Hungary) having an isomer shift equal to that of the CaSnO_3_ source matrix. Circular absorbers with a diameter of 15.5 mm were prepared by uniformly mixing approximately 100 mg of cellulose (as a filler) with either 14.2 mg of SP1a powder or 15 mg of SP1b and SP1c powders. The MossWinn 4.0 program (Institute for Nuclear Research (Atomki), Debrecen, Hungary) was used to analyze the spectra, assuming the thin absorber approximation.

UV–Vis reflectance spectra were recorded with a Shimadzu UV/VIS/NIR spectrometer, model UV-3600 (Shimadzu Corporation, Kyoto, Japan). The wavelength range used was from 600 to 200 nm.

### 2.6. Catalytic Measurements

The catalytic reduction of 4-nitrophenol (4-NP) to 4-aminophenol (4-AP) was investigated using UV–visible spectrophotometry in the presence of NaBH_4_ and the synthesized Pt-loaded samples. Before each measurement, the solutions containing 4-NP and NaBH_4_ were not purged with nitrogen. Immediately before each experiment, a fresh aqueous solution of NaBH_4_ was prepared to maintain the reducing efficiency. For the SP1a sample, 0.3 μmol of 4-NP (20 μL of a 0.015 M solution) was diluted with 2.7 mL of ultrapure water in a quartz cuvette, followed by the addition of 79.3 μmol of NaBH_4_ (20 μL of a 0.793 M solution) [19]. Samples SP1b and SP1c, because of their size, settled down before being able to act as catalysts. For better dispersion, samples SP1b and SP1c were prepared for catalytic measurements in the following manner: 0.3 μmol of 4-NP (20 μL of a 0.015 M solution) was diluted with 2.7 mL of 1% polyvinylpyrrolidone (PVP) solution in a quartz cuvette, followed by the addition of 79.3 μmol of NaBH_4_ (20 μL of a 0.793 M solution). Subsequently, 20 μL of a catalyst suspension (3 mg/mL in ultrapure water) was added and rapidly mixed using a micropipette. UV–visible spectra were recorded immediately after catalyst addition and monitored over time until the characteristic absorbance of nitrophenolate ions at 400 nm disappeared. The progress of the reaction and the formation of 4-AP were tracked by the emergence of a new absorption maximum at 300 nm.

The catalyst reusability was tested under the same experimental conditions in ten consecutive cycles. After each cycle, an additional 0.3 μmol of 4-NP was added to the cuvette and thoroughly mixed, followed by spectral recording, as described. To maintain a constant excess of reducing agent, 79.3 μmol of NaBH_4_ was also added before the fourth and seventh cycles. This approach ensured sufficient NaBH_4_ concentration throughout the experiment, allowing a reliable evaluation of catalyst stability and durability.

## 3. Results

### 3.1. XRD Results

**Figure 3:**
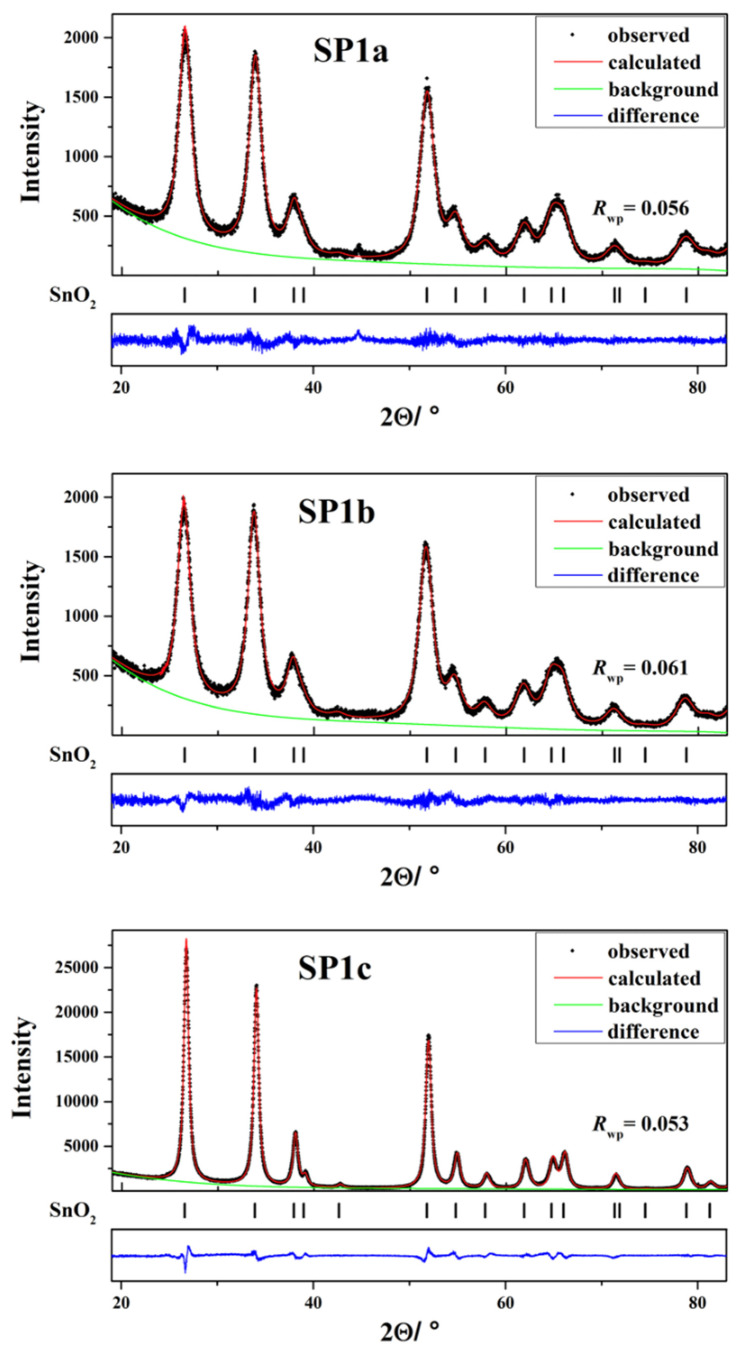
Rietveld refinements of samples SP1a, SP1b, and SP1c.

The XRD patterns of the synthesized supports and samples are shown in Figure S1, where only cassiterite was present. Figure 3 and Table 1 show the Rietveld refinement results, which confirm that all three Pt-loaded samples (SP1a, SP1b, and SP1c) crystallize in the cassiterite phase of SnO_2_. SP1a exhibits a relatively small crystallite size of 5.0 nm and a microstrain value of 5.0 × 10^–3^, illustrating a nanocrystalline structure with considerable lattice disorder. SP1b displays a similar crystallite size of 5.1 nm and a slightly reduced microstrain of 4.9 × 10^–3^, indicating a slight improvement in lattice order due to hydrothermal synthesis [20]. Conversely, sample SP1c, which was subjected to both hydrothermal treatment and subsequent annealing at 600 °C, has a significantly larger crystallite size of 13 nm and a significantly reduced microstrain of 1.4 × 10^–3^. This demonstrates considerable grain growth and improved crystallinity, consistent with high-temperature annealing.

### 3.2. STEM Results

**Figure 4:**
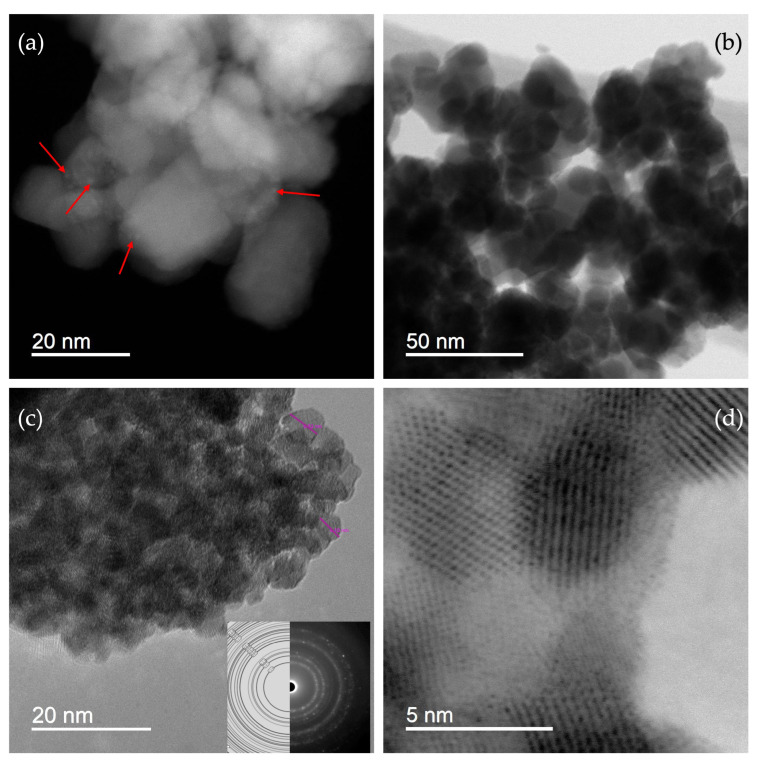
STEM DF image at high magnification, with arrows pointing towards PtOx nanoparticles (**a**); STEM BF image at high magnification (**b**); a high-resolution image with a SAED image in the inset; the powder patterns are indexed to SnO_2_ (cassiterite) (**c**); a high-resolution BF/STEM image of several SnO_2_-NPs with clearly visible lattice fringes (**d**).

STEM and SAED (selected area electron diffraction) results of sample SP1a are shown in Figure 4. Figure 4a shows the STEM dark field (DF) image at high magnification, with red arrows pointing to molecularly dispersed PtNPs, i.e., the small bright dots in the DF image corresponding to heavy elements (in this case, platinum). Figure 4b displays the STEM bright field (BF) image, while Figure 4c shows a high-resolution image of SnO_2_ with a SAED image in the inset, where the powder patterns are indexed to SnO_2_ (cassiterite). Figure 4d shows a high-resolution BF/STEM image with clearly visible lattice fringes.

**Figure 5:**
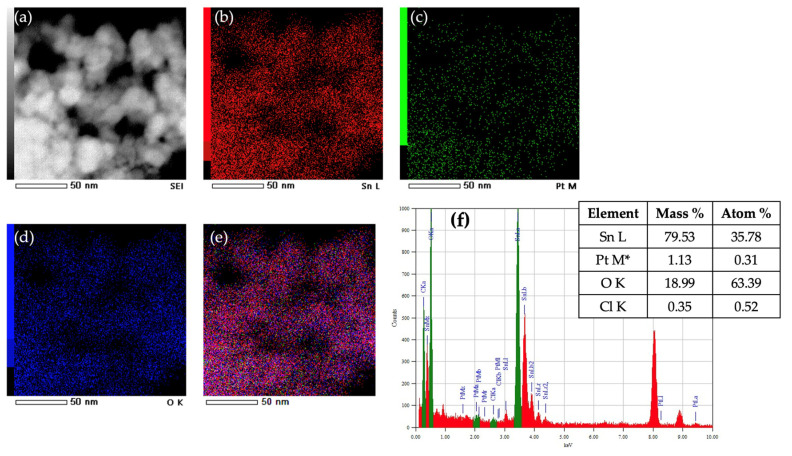
STEM image of the SP1a sample (**a**) and corresponding EDXS elemental mapping images of Sn L edge (**b**), Pt M edge (**c**), O K edge (**d**), and superposition of Sn L, Pt M, and O K edges (**e**). The EDXS spectrum in (**f**) confirms the presence of platinum and contains a small amount of chloride, as shown in the table. The asterisk * represents manually added Pt as a trace element.

Figure 5 presents the STEM-EDS elemental mapping and quantitative EDS analysis of the SP1a sample, providing further confirmation of the presence and distribution of platinum. Panel (a) shows a high magnification scanning electron image revealing well-defined SnO_2_ nanoparticles. Elemental mapping for Sn (b), Pt (c), and O (d) illustrates a uniform spatial distribution of all three elements in the sample. The overlay image (e) of Sn, Pt, and O shows homogeneous intermixing with no discernible platinum agglomerates, supporting the claim of molecularly dispersed oxidized platinum species. The quantitative EDS spectrum in panel (f) confirms the elemental composition with a platinum content of 1.13 wt% (0.31 at%), which is consistent with the intended loading of 1 mol%. The oxygen and tin content corresponds to the near stoichiometric SnO_2_, while the trace presence of chlorine (0.35 wt%) may suggest minor chloride residues from the H_2_PtCl_6_ precursor. The presence of oxidized PtOx species likely contributes to the diffuse appearance of platinum in the STEM micrographs. In contrast to metallic Pt nanoparticles, which typically appear as sharp, high-contrast dots due to their electron density, these oxygen-coordinated species reduce the local contrast so that individual Pt centers appear blurred or indistinct. This effect explains the absence of clearly defined Pt particles in the STEM-DF image of Figure 4a, although element mapping and EDS confirm their presence.

**Figure 6:**
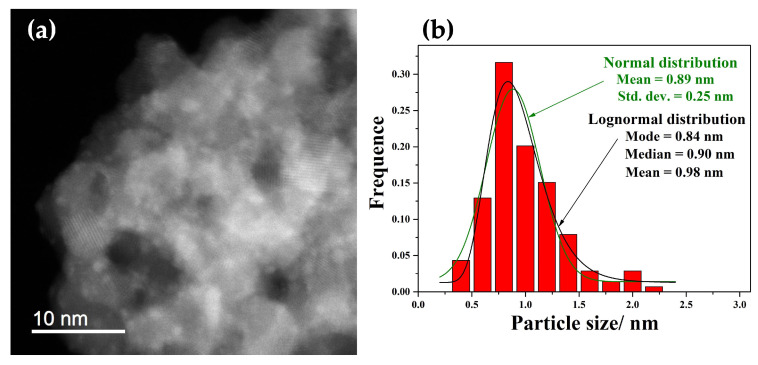
STEM DF image at high magnification of the sample with 10 mol % Pt (the SP10a sample) (**a**), and size distributions of the platinum particles calculated using the normal and lognormal functions (**b**) from the image in (**a**).

To resolve the uncertainty regarding the identification of platinum in the STEM micrograph shown in Figure 4a—where the low platinum loading (1 mol%) made it difficult to assign the observed contrast variations to platinum with certainty—we synthesized an additional sample with a much higher platinum content (10 mol% Pt, sample SP10a). This higher loading allowed a clearer visualization of the platinum species in the STEM images, as can be seen in the micrograph shown in Figure 6. The size distributions of the platinum particles were calculated using the normal and lognormal functions from the image in (6a). Figure S2 shows the same STEM image of the SP10a sample, with line markers (added using the ImageJ 1.53 software (Bethesda, MD, USA) indicating the measured dimensions of the platinum nanoparticles. Figure S3 shows the STEM image of sample SP10a (a) and the corresponding EDXS elemental images. Compared to the SP1a sample (Figure 5), a clear increase in the Pt concentration can be seen in the SP10a sample loaded with 10 mol% Pt, while the increase in the Cl concentration is not visible despite the addition of Pt in chloride form as H_2_PtCl_6_.

### 3.3. Thermal Analysis

**Figure 7:**
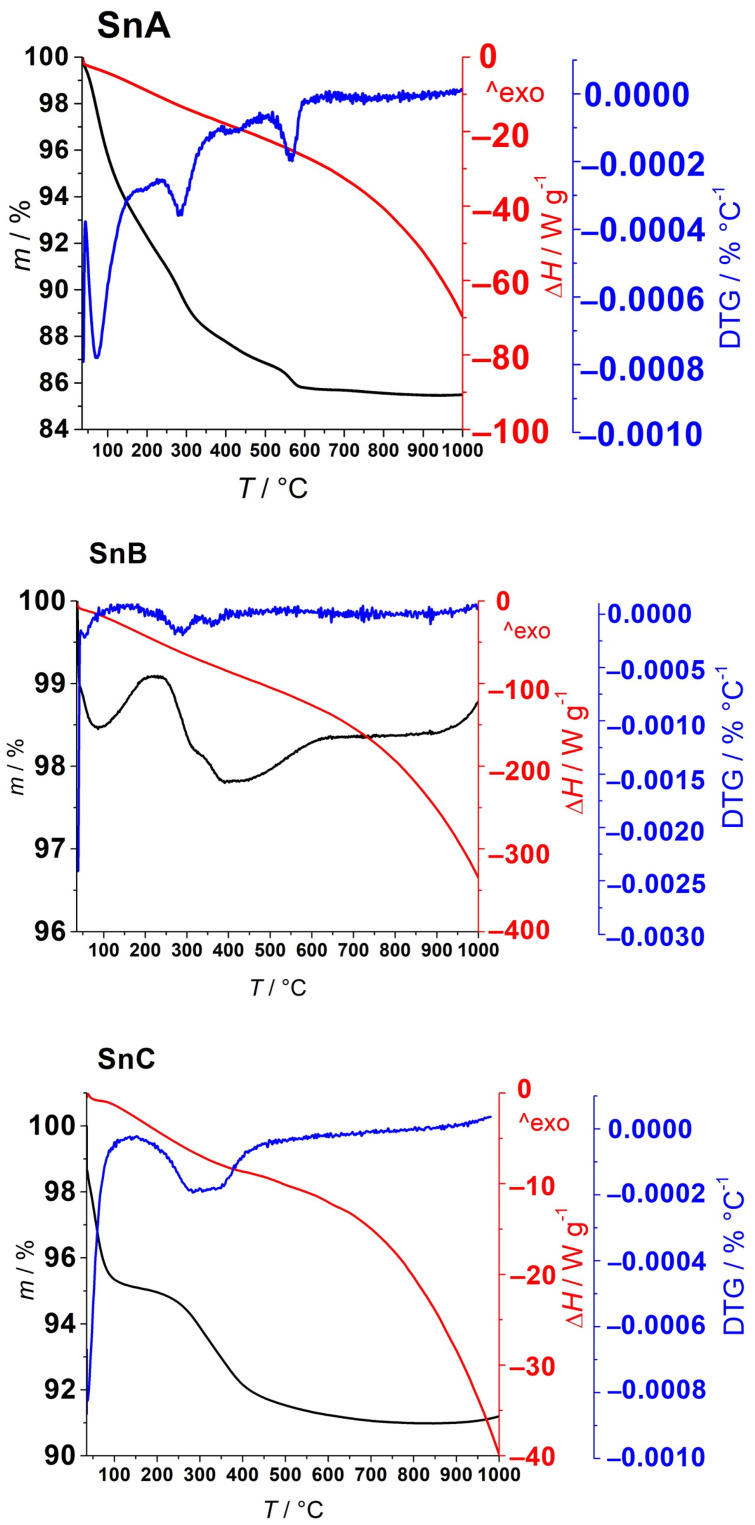
TGA-DTG-DSC thermograms (black–blue–red curves) of SnA (**top**), SnB (**middle**), and SnC (**bottom**) supports recorded in nitrogen atmosphere up to 1000 °C.

Thermogravimetric analysis and differential scan calorimetry of the three supports were performed to investigate their thermal stability and decomposition behavior. Figure 7 shows the following results: differential scanning calorimetry (DSC; red curves), thermogravimetric analysis (TGA; black curves), and derivative thermogravimetry (DTG; blue curves).

The thermal behavior and structural evolution of the three supports, SnA–C, reflect the influence of synthetic and post-synthetic treatments on their physicochemical properties, which are crucial for their function as catalyst supports. Hydrothermal synthesis significantly improves the initial crystallinity of SnO_2_ and provides a better ordered oxide framework compared to purely sol–gel- or precipitation-derived materials. Subsequent annealing at higher temperatures, in this case at 600 °C, further improves structural integrity by removing residual hydroxyl groups and volatile impurities, resulting in a material with greater thermal and chemical stability. The most notable exothermic transition between 500 and 600 °C indicates structural rearrangement, suggesting that the support has transitioned into a thermally stable phase [21].

The thermal analysis of SnA and SP10a (Figure S4) illustrates the influence of platinum intercalation on the stability of the SnO_2_ matrix. SnA shows clear mass loss steps in the TGA and sharp DTG peaks below 600 °C, which correspond to the removal of physisorbed water and hydroxyl groups. In contrast, SP10a exhibits a more gradual mass loss with broader DTG features, indicating smoother thermal transitions and improved thermal stability. The lower overall mass loss and suppressed DTG peaks in SP10a suggest that the presence of 10 mol% Pt alters the surface chemistry, possibly through strong metal–support interactions and partial Pt reduction, resulting in a more thermally robust catalyst. The presence of 10 mol% Pt on the SnO_2_ support leads to more pronounced thermal transitions in the DTG profile due to the surface-bound hydrolyzed Pt species, especially Pt–O, Pt–Cl, and Pt–OH complexes. The formed PtOx species do not simply incorporate into the SnO_2_ lattice—instead, they anchor to the surface and interact strongly with Sn hydroxyl groups, leading to localized structural rearrangements and additional desorption or decomposition processes during heating.

### 3.4. BET Nitrogen Adsorption–Desorption Isotherm and Pore Volume

**Figure 8:**
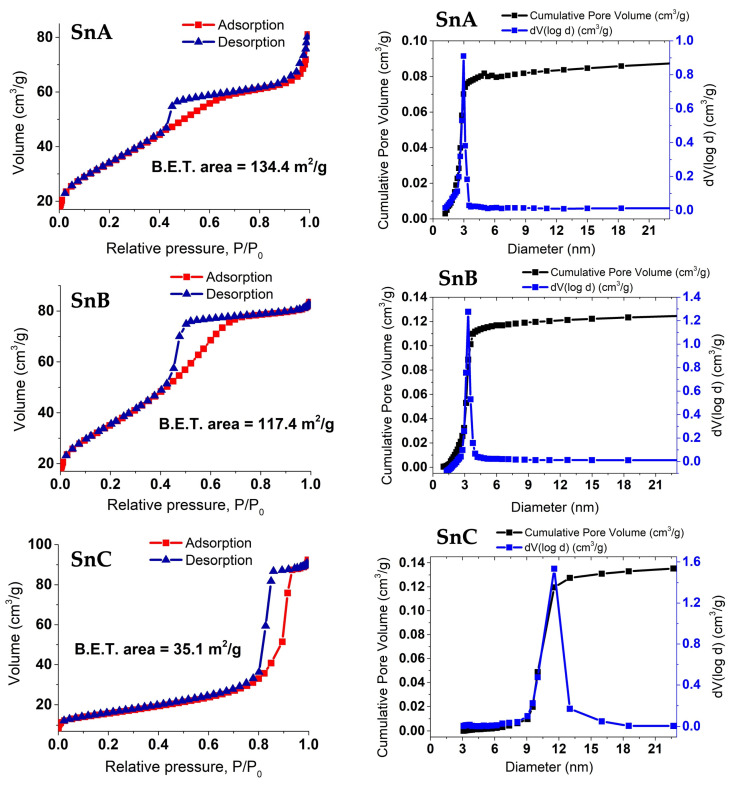

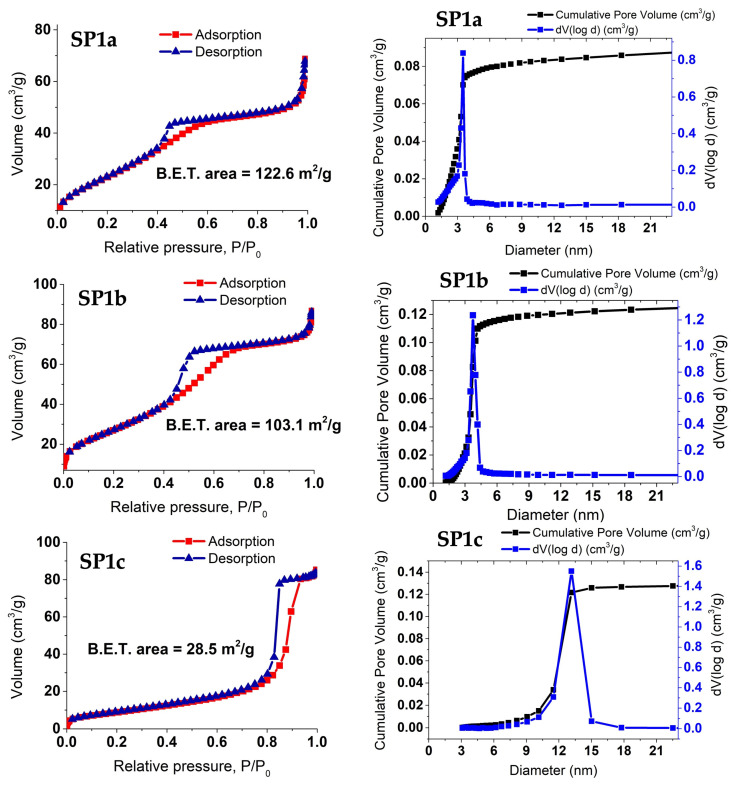
Isotherms of adsorption of nitrogen (N_2_) (red line, squares) and desorption (blue line, triangles) for the SnA–C and SP1a–c supports, together with the determined BET surface areas. The corresponding pore volume distribution provides information about the porosity of the material.

Figure 8 shows the nitrogen (N_2_) adsorption–desorption isotherms and corresponding pore size distribution profiles for the SnO_2_-based supports labeled SnA, SnB, and SnC, along with their Pt-modified counterparts SP1a, SP1b, and SP1c. All samples exhibit type IV isotherms with H1-type hysteresis (SnC and SP1c) and H2-type hysteresis loops (SnA, SnB, SP1a and SP1b), characteristic of mesoporous materials (pores 2–50 nm in diameter) where capillary condensation occurs at intermediate relative pressures [22]. The H2-type hysteresis loops are generally associated with complex pore architectures, such as ink-bottle-shaped pores or disordered mesoporous networks with non-uniform connectivity and pore sizes, often arising from non-rigid aggregates of plate-like or spheroidal particles [23].

### 3.5. XPS Results

**Figure 9:**
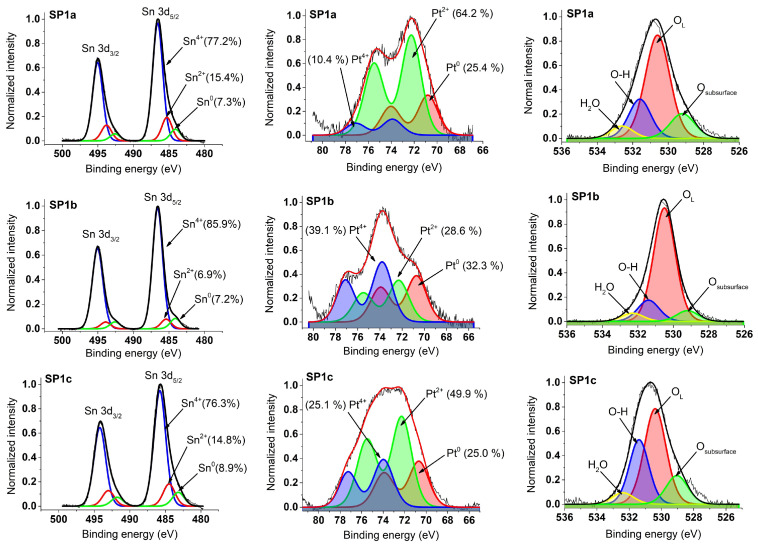
XPS spectra of samples SP1a–c, measured around Sn 3d (**left panel**), Pt 4f (**middle panel**), and O1s (**right panel**) core levels.

In the left panel of Figure 9, the plots show photoemission spectra comprising the Sn 3d core levels in the samples SP1a–c. These spectra show three spin-orbital doublet patterns corresponding to the different oxidation states of tin, namely Sn^0^, Sn^2+^, and Sn^4+^. The dominant signal originates from Sn atoms in the Sn^4+^ state (SnO_2_), as evidenced by the Sn 3d_5/2_ peak with the binding energy (BE) of around 486 eV and the 3d_3/2_ peak shifts by 8.5 eV to a higher BE.

The middle of Figure 9 shows the Pt 4f photoemission curves of the Pt-loaded samples. These spectra are numerically deconvoluted with two or three doublets assigned to the platinum oxidation states, i.e., Pt^0^, Pt^2+^, and Pt^4+^. The energy positions of the Pt 4f_7/2_ peaks are around 70.7 eV (Pt^0^), 72.3 eV (Pt^2+^), and 74.0 eV (Pt^4+^). It is noteworthy that the energy separation between the Pt 4f_7/2_ and Pt 4f_5/2_ components is consistent at about 3.3 eV for all Pt oxidation states, which agrees well with existing data on platinum oxides from the previous literature.

The fitted Pt 4f and Sn 3d spectra allow for the determination of the peak positions and relative contributions (%) of Pt^4+^, Pt^2+^, Pt^0^, Sn^4+^, Sn^2+^, and Sn^0^ in the synthesized samples. Full details of these results, including numerical values extracted from the deconvolution, are given in Supplementary Table S2. Table 2 contains the XPS-derived average oxidation states (AOS) of platinum (Pt) and tin (Sn) obtained in this study.

Four oxygen species are identified in the O 1s photoemission spectra (Figure 9, right panel) of samples SP1a, SP1b, and SP1c, namely lattice oxygen (O_L_) at ~530.5 eV, hydroxyl groups (O–H) at ~531.5 eV, physisorbed water (H_2_O) at ~532.5 eV, and subsurface oxygen at ~529.0 eV. The differences in signal contribution reflect the influence of the synthesis conditions on the surface composition, defect structure and oxygen-related species relevant to the catalytic behavior [24].

XPS analysis was also performed for the sample with a higher Pt loading (SP10a), and the results are given in Figure S5.

### 3.6. Raman Spectroscopy Results

**Figure 10:**
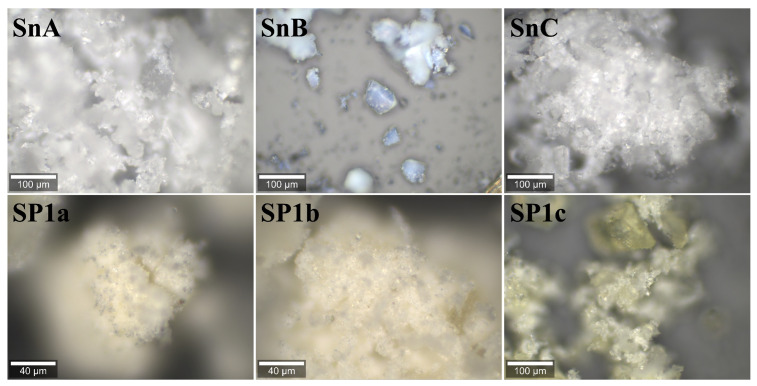
Visual representation of the supports and samples that were analyzed using Raman spectroscopy and shown in Figure 11.

**Figure 11:**
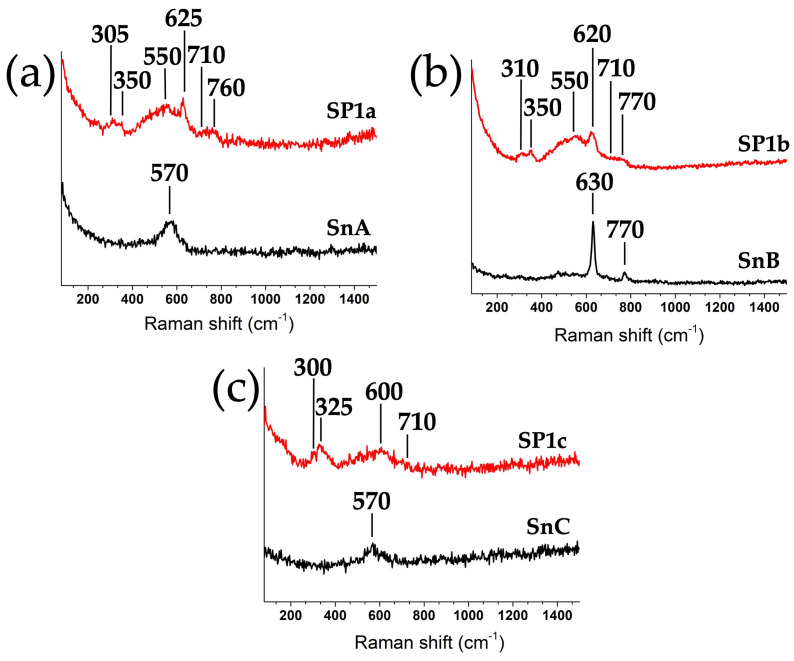
Raman spectra (532 nm excitation) for supports and samples: (**a**) SnA and SP1a, (**b**) SnB and SP1b, and (**c**) SnC and SP1c.

Raman spectroscopy was used to analyze the structural features of the SnO_2_ supports and the platinum-loaded samples (see Figure 10). The spectra presented in Figure 11 show that after the incorporation of 1 mol% Pt, all samples exhibit significantly broader Raman bands, indicating the introduction of surface and lattice disorder. This broadening indicates that platinum species interact strongly with the SnO_2_ surface, leading to local symmetry perturbations and possibly creating surface defects. In particular, a number of broad and weak bands in the 250–350 cm^−1^ range appear in all Pt-loaded samples [25]. These bands are not predicted by group theory for the ideal cassiterite structure and are instead attributed to either surface-induced disorder or vibrational properties of the Pt species. In particular, the band at 330 cm^−1^ is associated with Pt–Cl stretching vibrations likely originating from hydrolyzed Pt complexes, which was confirmed by the increased intensity of this band in the SP10a sample with 10 mol% Pt (see Figure S8). The weak band between 550–600 cm^−1^ is attributed to Pt–O vibrations and gradually disappears after annealing, suggesting enhanced crystallinity and the removal of amorphous or hydrous surface species. The characteristic SnO_2_ lattice vibrations are also observed, i.e., the symmetric Sn–O stretching mode A_1_g around 620–635 cm^−1^ and the asymmetric mode B_2_g around 745–760 cm^−1^, both of which are slightly distorted in the Pt-containing samples SP1a and SP1b, indicating interactions between Pt species and the SnO_2_ matrix [26].

### 3.7. ^119^Sn Mössbauer Spectroscopy Results

**Figure 12:**
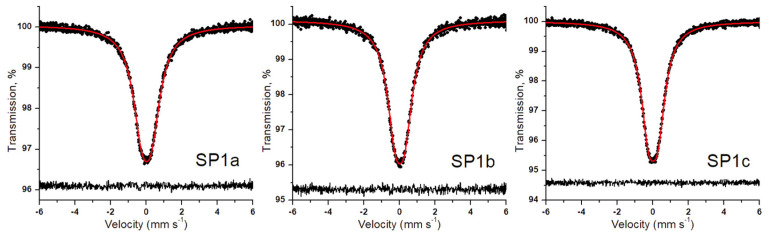
Room-temperature ^119^Sn Mössbauer spectra (dots) of the SP1a–c samples along with the envelope (solid line) of the Lorentzian quadrupole doublet fitted. The fit residual is shown below the spectra.

The ^119^Sn Mössbauer spectra of the SP1a, SP1b, and SP1c samples are displayed in Figure 12. The main feature of the spectra is a broad absorption peak centered at around 0 mm s^−1^, typical for the 4+ oxidation state of tin in SnO_2_. Absorption signals attributable to stannous phases were not observed in the spectra. The broad peak can be decomposed into a symmetric quadrupole doublet of Lorentzians, with the nonzero quadrupole splitting reflecting the asymmetry of the SnO_6_ octahedra in SnO_2_ [27]. Table 3 contains all the ^119^Sn Mössbauer parameters derived from the spectra. The isomer shift of the samples is above zero, suggesting a slightly higher electron density at the 5s orbital of Sn^4+^ than in our SnO_2_ reference. The quadrupole splitting and line width parameters of the sample with the highest crystallinity (SP1c) lie close to those observed earlier for microcrystalline SnO_2_ [27], and they are also consistent with recent results obtained on Pt/SnO_2_ catalysts prepared via microwave-assisted synthesis [13]. As shown in Figure S9, the value of the quadrupole splitting and the line width both increase with decreasing crystallinity of the samples. A similar trend is observed for the isomer shift parameter as well (Table 3). Comparison of the minimum transmission levels of the spectra in Figure 12 also reveals a decreasing tendency of the Mössbauer effect with decreasing crystallinity of the samples. By defining the quantity of “absorption strength” as the Mössbauer spectral area normalized to the baseline and the applied sample mass (to account for the slightly lower mass applied in the case of SP1a), and expressing it relative to sample SP1c, the tendency can be clearly observed, as shown in Figure S10. This indicates that the vibrational state of Sn^4+^ ions changes with the degree of crystallinity, resulting in lower recoilless fractions in samples with lower crystallinity.

### 3.8. Catalytic Measurements

**Figure 13:**
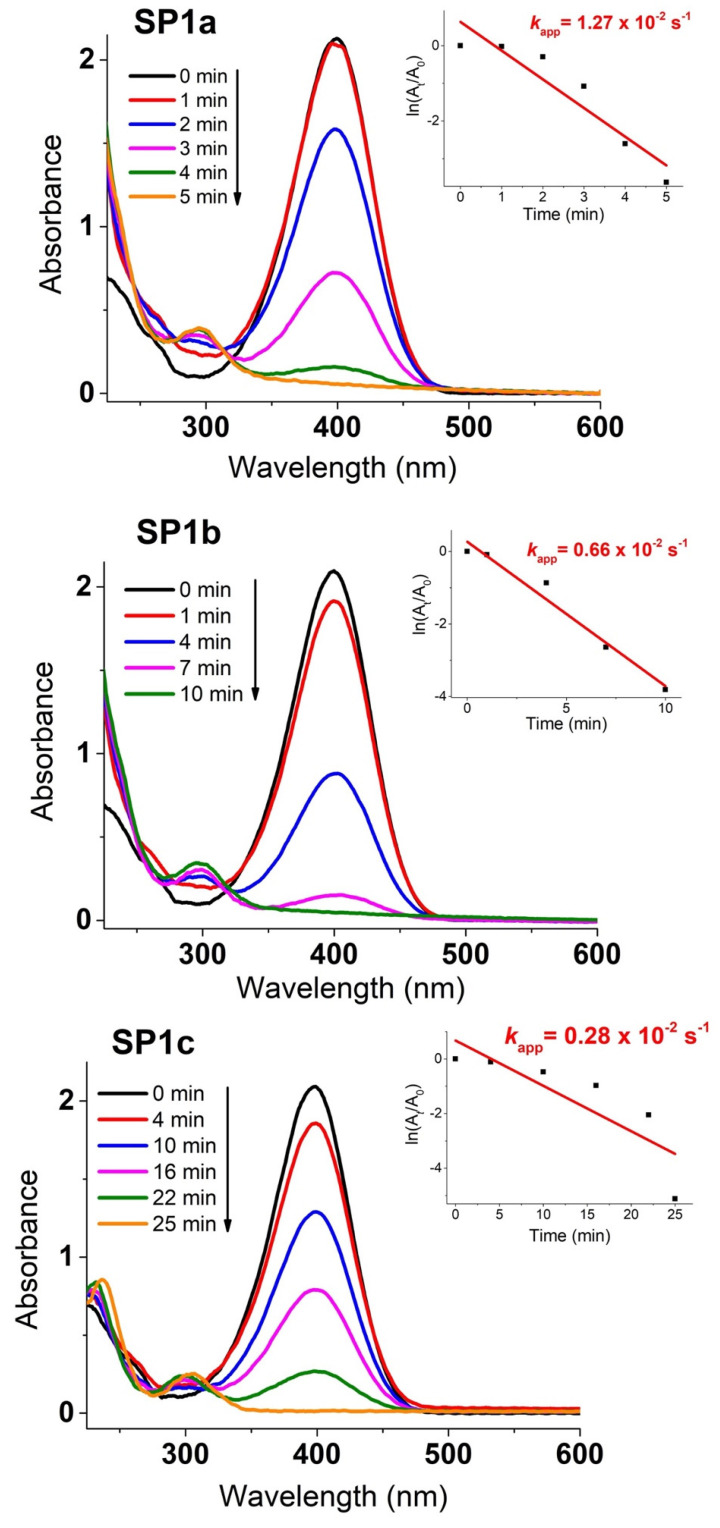
Time-dependent catalytic reduction process of 4-nitrophenol (4-NP) to 4 aminophenol (4-AP) using platinum-decorated SnO_2_ samples (SP1a, SP1b, and SP1c). The insets show the ln(A*_t_*/A_0_) plot versus the reaction time and the calculated values of the rate constants (*k*_app_ in s^−1^) derived from the slopes of the linear segments.

Figure 13 shows the time course of the catalytic reduction of 4-nitrophenol (4-NP) to 4-aminophenol (4-AP) with excess NaBH_4_ over time, utilizing platinum decorated on a SnO_2_ support. The insets showcase the plot of ln(A*_t_*/A_0_) versus reaction time. The values of the rate constant kapp (s^−1^) shown were derived from the slopes of the linear segments based on the following pseudo-first-order kinetic equation [28]:

ln(*C_t_*/*C*_0_) = ln(A*_t_*/A_0_) = –*k*_app_ × t, (1)

The SnA support, without any added platinum, is entirely inactive for the catalytic reduction of 4-NP to 4-AP (refer to the Supplementary Materials). In contrast, the platinum-loaded samples exhibit strong catalytic activity. Among them, the as-synthesized platinum-loaded SnO_2_ (sample SP1a) emerges as the most catalytically potent for 4-NP to 4-AP reduction, marked by a *k*_app_ rate constant of 1.27 × 10^−2^ s^−1^. The as-synthesized sample (SP1a) shows a better dispersion of the PtNPs and a higher catalytic activity than the autoclaved samples (SP1b and SP1c), for which a PVP coating of the samples is required for better dispersion in the solution [29]. This catalytic efficiency is reflected in the decreasing absorbance at 400 nm, associated with the disappearance of 4-nitrophenolate ions, and the simultaneous increase at 300 nm, indicating the formation of 4-aminophenol [30,31] (see Figures S11–S14).

**Figure 14:**
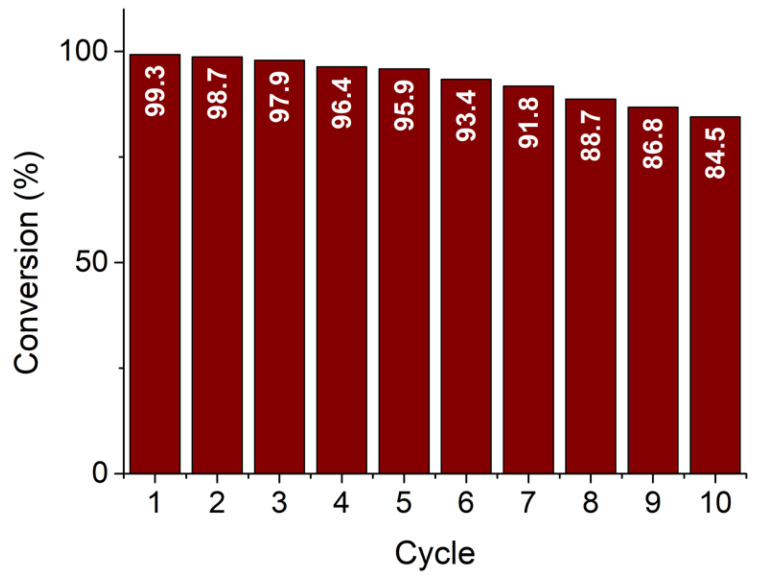
Reusability test of the SP1a sample for 10 cycles.

The recyclability (reusability) test was performed for the SP1a sample with the highest catalytic activity. The test was performed in the same way as the other catalytic measurements for a total of 10 cycles. As can be seen in Figure 14, the SP1a sample is very robust and exhibits very high efficiency in reducing 4-NP to 4-AP even after 10 cycles.

## 4. Discussion

In previous studies, platinum-based catalysts were synthesized on various metal oxides, such as Fe_2_O_3_, SnO_2_, and MnO_2_, by thermal or mechanochemical methods, often using organometallic precursors and high-temperature post-treatments in controlled atmospheres. Platinum-doped SnO_2_, for example, was produced by wet impregnation of Pt(acac)_2_ in toluene, followed by ball-milling and annealing in argon and oxygen atmospheres at 400 °C [32]. Another approach was to synthesize MnO_2_ nanostructures by microwave-assisted hydrothermal treatment, followed by solvent evaporation and annealing after mixing with Pt(acac)_2_ in toluene to achieve different Pt loadings [33]. While these methods are effective, they require either high temperatures, long processing times, or organic solvents, which can limit scalability and environmental compatibility.

The method we used in our previous work [13] was the microwave-assisted hydrothermal synthesis of Pt-doped SnO_2_ materials by direct coprecipitation of H_2_PtCl_6_ and SnCl_4_ at very low pH, followed by ammonia-induced precipitation, microwave treatment at 230 °C, and calcination at 400 °C. This process provided better control over the Pt doping concentration with molar ratios ranging from 0 to 15 mol% and aimed to produce well-dispersed Pt species within the SnO_2_ matrix. Although this route is more aqueous in nature, it still required high-temperature microwave synthesis and post-synthetic annealing to ensure oxide formation and phase purity.

In contrast, the present work presents an aqueous low-temperature synthesis of Pt/SnO_2_ catalysts based on a chloride-free precursor approach, in which SnCl_4_ is first purified by anion exchange and platinum is introduced as H_2_PtCl_6_ under mild conditions without the use of external reducing agents or organic solvents. This synthesis strategy eliminates the need for the high-temperature decomposition of organometallic complexes and enables the formation of catalytically active PtOx species (Pt^2+^ and Pt^4+^) at room temperature. Moreover, the approach emphasizes strong metal–support interactions on hydroxyl-rich SnO_2_ surfaces, which not only stabilize the platinum species but also contribute to their catalytic activity, as demonstrated in the reduction of 4-nitrophenol. This work, therefore, provides a more sustainable and controllable route to platinum-based catalysts compared to previous methods, while expanding the mechanistic understanding of oxidized Pt species in heterogeneous catalysis.

The structural, morphological and surface chemical properties of the supports and samples were systematically analyzed by XRD with Rietveld refinement, STEM imaging, TGA/DSC/DTG analysis, BET surface analysis, XPS, Raman spectroscopy, and ^119^Sn Mössbauer spectroscopy. The catalytic activity of the samples was evaluated using the model reduction of 4-nitrophenol to 4-aminophenol in aqueous NaBH_4_ under ambient conditions and monitored by UV–Vis spectroscopy.

The Rietveld refinements (Figure 3 and Table 1) provide detailed structural comparisons between the three SnO_2_-based samples—SP1a, SP1b, and SP1c—which were each subjected to increasingly intense thermal treatment. SP1a, which was synthesized only by removing chloride ions through anion exchange, shows broad and low-intensity diffraction peaks, indicating low crystallinity and a nanocrystalline structure with high lattice disorder. This is to be expected for a material that has not undergone hydrothermal or thermal processing. SP1b, which was hydrothermally treated at 180 °C for 24 h, shows narrower and more intense diffraction peaks, indicating a significant improvement in crystal quality and order in the SnO_2_ lattice [34]. The most pronounced improvement in crystallinity is observed in SP1c, which was subjected to both hydrothermal treatment and subsequent annealing at 600 °C. This sample exhibits the sharpest and most intense peaks as well as the narrowest full width at half maximum (FWHM), indicating larger crystallite sizes and minimal microstrain [35]. These results are in excellent agreement with complementary data from XPS and Raman spectroscopy, which also indicate a higher degree of crystallinity and fewer surface defects. The degree of SnO_2_ crystallinity influences the local environment of Sn^4+^ ions, as evidenced by variations in the isomer shift and quadrupole splitting values observed in the ^119^Sn Mössbauer spectroscopy. Taken together, these structural differences help to explain the different catalytic activity and thermal stability of the three samples. The quality of the Rietveld fit, as indicated by the *R*_wp_ values, remains acceptable for all samples with values between 0.053 and 0.061, confirming the reliability of the refinements. These results show that crystallinity and structural coherence gradually improve with increasing thermal treatment.

STEM-EDXS mapping and high-resolution imaging (Figure 4 and Figure 5) confirm the homogeneous distribution of the Pt species on the SnO_2_ support. In particular, the Pt particles are molecularly dispersed, with no signs of agglomeration or large clusters, supporting the hypothesis that platinum is present in an oxidized and highly dispersed form.

We chose two platinum loadings—1 mol% and 10 mol%—to achieve a balance between catalytic relevance and structural characterization. The 1 mol% Pt loading was chosen as a representative concentration commonly used in catalysis to ensure sufficient dispersion of the active sites while minimizing platinum consumption. However, due to the high dispersion and low atomic contrast of the PtOx nanoparticles at this concentration, direct imaging and confirmation of their presence using STEM proved to be difficult. To overcome this limitation and clearly confirm the localization and morphology of the Pt species on the SnA support, a second sample was synthesized with 10 mol% Pt. The histogram in Figure 6 displays the size distribution of PtOx NPs obtained from STEM micrographs with fits based on normal and lognormal distribution models. The normal distribution fit yields a mean particle size of 0.89 nm and a standard deviation of 0.25 nm, indicating a narrow and uniform distribution of particle size. The lognormal distribution, which better reflects skewed distributions common in nanoparticle systems, yields a mode of 0.84 nm, a median of 0.90 nm, and a mean of 0.98 nm. The slight right skew of the histogram supports the appropriateness of the lognormal model and reflects the presence of some larger particles. Overall, the data confirm that the PtNPs have a narrow and well-controlled size distribution centered below 1 nm, consistent with high dispersion on the support, which is advantageous for catalytic applications due to the larger surface area and number of accessible active sites [23].

SnA exhibits the highest nitrogen uptake and BET surface area (134.4 m^2^/g), indicating a well-developed mesoporous structure with a narrow pore size distribution centered around 3.2 nm. This high mesoporosity results from the interparticle spaces between uniform 5 nm nanocrystalline cassiterite particles formed via a low-temperature, chloride-free aqueous precipitation route. Specifically, SnA was prepared via an anion exchange route, wherein an aqueous solution of SnCl_4_ was subjected to chloride removal using an anion exchange resin. This process leads to the immediate (in 20-30 min) appearance of a milky-white colloidal suspension, indicative of the rapid formation of Sn(IV)-oxo or hydroxide clusters due to extensive hydrolysis and subsequent olation/oxolation reactions, especially in the absence of chloride, which normally stabilizes Sn^4+^ in solution. The primary hydrolysis step can be represented with Reaction (1), followed by the condensation reactions in Reaction (2):

Sn^4+^ + x H_2_O ⇌ Sn(OH)_x_^(4−x)+^ (R1)

Sn(OH)_x_ ⇌ Sn-O-Sn + H_2_O (R2)

This leads to the formation of small, highly hydrated tin–oxo clusters, which then aggregate into nanocrystalline cassiterite (SnO_2_), as confirmed by XRD and STEM. Due to the low temperature and aqueous environment, crystal growth is kinetically limited, resulting in crystallite sizes of ~5 nm. Importantly, these nanocrystals do not coarsen significantly due to the absence of thermal treatment, preserving high dispersion and interparticle voids, thus forming a mesoporous network without the need for templating agents. The H2-type hysteresis suggests constricted or “bottleneck” pores due to the random packing of nanocrystals. SnB also displays a mesoporous profile with a slightly reduced surface area of 117.4 m^2^/g and its pore size distribution peaking around 3.4 nm, indicating a similar but marginally wider pore network after autoclaving. In contrast, SnC, which was annealed at 600 °C, exhibits significantly lower nitrogen uptake and a reduced BET surface area of 35.1 m^2^/g, reflecting a much less developed mesostructure. Its pore size distribution is broader and shifted toward larger diameters (~8.5–9 nm), indicating a less uniform and partially collapsed or sintered porous structure due to the thermal treatment.

The introduction of Pt, through hydrolysis of H_2_PtCl_6_ at 40 °C, generally leads to a decrease in specific surface areas and altered pore volumes across all supports, while maintaining their type IV/H2 characteristics. SP1a (Pt on SnA) displays a BET surface area of 122.6 m^2^/g, slightly lower than SnA (134.4 m^2^/g), indicating partial pore blockage or surface coverage by highly dispersed 0.85 nm PtOx clusters (“faint stains” or patches) as observed by STEM. The isotherm maintains the H2-type hysteresis, with a subtle narrowing in the desorption branch suggesting a more constrained mesopore network, while the pore size distribution remains centered around 3.2 nm. The Pt precursor (H_2_PtCl_6_) was added without chloride removal, introducing residual Cl^−^ into the system. The strong interaction of H_2_PtCl_6_ with the hydrated SnO_2_ surface, combined with ambient oxidation conditions, likely leads to the formation of highly dispersed hydrated PtOx species via hydrolysis reactions on the surface. For SP1b (Pt on autoclaved SnB), the surface area reduces to 103.1 m^2^/g compared to SnB (117.4 m^2^/g), consistent with Pt-induced partial pore coverage. The H2-type hysteresis remains visible but is narrower and shifted toward lower relative pressures, indicating smaller or more constricted pores, possibly due to structural rearrangement during hydrothermal treatment and subsequent Pt deposition, with the pore size distribution still centered around 3.2–3.4 nm. The strong interaction with the support promotes well-dispersed PtOx even with residual chloride. SP1c (Pt on annealed SnC) shows the most pronounced changes, with its surface area dropping significantly to 28.5 m^2^/g (from 35.1 m^2^/g for SnC). The isotherm reveals a broader hysteresis loop (H1-type for SnC and SP1c) at higher relative pressures (P/P_0_ > 0.6), indicating a more disordered and less uniform mesoporous system due to crystallite sintering and grain growth during annealing at 600 °C. The pore size distribution shifts dramatically to larger diameters (10–15 nm), reflecting the collapse of smaller pores and partial destruction of mesoporosity. Consequently, the PtOx dispersion occurs primarily at the external surface due to the largely lost internal mesopores. In summary, the synthetic conditions critically influence the mesoporous texture, with SnA and SnB retaining high surface areas and uniform mesoporosity post-Pt incorporation, making them suitable for active species dispersion. The observed textural changes upon Pt incorporation, including the subtle reduction in surface area and potential pore narrowing, reflect the effective dispersion of ultrasmall PtOx domains on the SnO_2_ supports.

From the combined characterization results, we conclude that platinum in the SP10a sample is predominantly present in the form of surface-anchored PtOx species with the presence of Sn-O-Sn, Pt-O-Pt, Pt–Cl, Pt–OH, and, most importantly, Pt-O-Sn bonds on the surface of SnO_2_ catalyst. Although the SP10a sample was synthesized with a significantly higher Pt loading (10 mol%) compared to SP1a (1 mol%), STEM-EDS analysis (Figure S3) shows no increase in chloride content, suggesting that the chloride is not freely dispersed but rather is coordinated to the Pt species. Thermal analysis of SP10a (Figure S4) shows a more gradual and prolonged mass loss profile than the chloride-free SnA support, indicating the presence of strongly bound hydrolyzed Pt complexes. This is confirmed by Raman spectroscopy (Figure S8), where characteristic bands are observed in the 300–330 cm^−1^ range, and are typically associated with the Pt–Cl vibrational modes. The Raman band at 710 cm^−1^ strongly suggested the presence of Pt-O-Sn surface bonds, thus confirming strong SnO_2_/PtOx interactions. In addition, XPS analysis (Figure 9 and Figure S5) confirms the predominance of PtOx platinum species, particularly Pt^2+^ and Pt^4+^, and shows a strong signal corresponding to hydroxyl groups, consistent with Pt–OH surface coordination. Taken together, these results suggest a system in which platinum is immobilized on SnO_2_ by stable PtOx species instead of forming mostly metallic nanoparticles.

Importantly, despite the mild synthesis conditions—room temperature and no external reducing agents—the XPS analysis revealed the presence of metallic platinum (Pt^0^) on the surface of the Pt/SnO_2_ catalysts. In the sample with 1 mol% Pt loading, about 25% of the Pt was present in metallic state, while in the sample with 10 mol% Pt, the Pt^0^ content increased to about 35% (Figure S5). Although this observation may seem counterintuitive since H_2_PtCl_6_ is normally thermally reduced at ≥300 °C, several factors may explain the spontaneous partial reduction of Pt^4+^ under our synthesis conditions. First, the SnO_2_ support (SnA) synthesized at room temperature contains a considerable number of surface hydroxyl groups (OH form) due to the use of an anion exchange resin. These surface –OH groups, which are coordinated to Sn^4+^ centers, can act as mild reducing agents. Their involvement in redox reactions is already known, especially in systems with strong metal–support interactions [36]. The following simplified redox reaction illustrates the possible mechanism:

2 Pt^4+^ + 2 OH^−^ → Pt^0^ + Pt^2+^ + H_2_O + ½ O_2_ (R3)

The higher concentrations of starting materials appear to favor partial autoreduction, likely due to increased local concentration and enrichment of Pt species, which may lead to local reduction through disproportionation or surface-mediated pathways during mixing and drying. Furthermore, the minor contribution of the photoreduction of Pt^4+^ cannot be excluded.

There is a direct correlation between the degree of thermal treatment and Raman spectral features (Figure 11) and XPS oxygen speciation (Figure 9, right panel). These two spectroscopies provide complementary insight into the defect structure of metal oxide materials. SP1a exhibits a broad, weak A_1g_ peak near 630 cm^−1^, indicating low crystallinity and high structural disorder, consistent with a SnO_2_ phase that has not been thermally treated. The presence of additional low-frequency scattering likely reflects a high density of surface defects and oxygen vacancies formed during ion exchange and precipitation without a subsequent ordering step. This was confirmed by the XPS spectrum measured around the O1s core levels, as the sample has a significant amount of water (H_2_O) and surface hydroxyl groups (O–H). SP1b shows a better defined A_1g_ Raman mode with reduced baseline noise, which is expected from a hydrothermal step facilitating the restructuring of the SnO_2_ framework [25]. Improved structural order with maintained surface reactivity is evident from the increase in lattice oxygen (O_L_) and the slight decrease in H_2_O and O–H in the XPS spectrum. SP1c displays the sharpest and most intense A_1g_ peak, confirming that annealing at 600 °C promotes high crystallinity and the removal of residual hydroxyl groups.

While both SnB and SnC were subjected to hydrothermal treatment, the additional thermal annealing at 600 °C in SnC promotes grain growth and surface tension relaxation, which may reduce the efficiency of Raman scattering for certain vibrational modes and broaden the bands due to increased phonon–phonon interactions or reduced phonon confinement. In contrast, SnB has a relatively high degree of crystallinity with moderate crystallite size and higher defect density, e.g., oxygen vacancies and hydroxyl groups on the surface, which can localize vibrational modes and increase Raman activity. This explains why SnB has sharper and more intense bands, especially for the A_1_g and B_2_g modes. The shift in Raman band positions between samples is primarily attributed to differences in crystallite size, lattice strain, and local bonding environment. These shifts are consistent with the phonon confinement model, where smaller crystallites (as in SnA and SnB) lead to slight blue or red shifts in peak positions due to the relaxation of selection rules.

The detection of Sn^2+^ and Sn^0^ species via XPS (Figure 9, left panel), based on Sn 3d core level analysis, contrasts with the absence of their signal in the ^119^Sn Mössbauer spectra, indicating that these species are confined to the surface of the SnO_2_ particles. Consequently, the average tin oxidation states reported in Table 2 primarily reflect the surface composition of SnO_2_. The interaction between Pt^2+^/Pt^4+^ species and the SnO_2_ support, a reducible n-type semiconductor, may facilitate electron transfer during the reaction, enabling catalytic function without the need for metallic nanoparticles.

Among the three catalysts, SP1a, prepared without thermal treatment, exhibited the highest specific surface area and the smallest average pore diameter (Figure 8), as well as the best catalytic activity (*k*_app_ = 1.27 × 10^−2^ s^−1^) and reusability, as it maintained a conversion of over 84% after ten cycles (Figure 13 and Figure 14). The observed catalytic activity despite the absence of metallic Pt^0^ underlines the functionality of the oxidized Pt species. The Pt^2+^ and Pt^4+^ species anchored to SnO_2_ appear to facilitate electron transfer from the reducing agent (NaBH_4_) to 4-NP via the support. This is likely due to the reducible, n-type semiconducting nature of SnO_2_, which can mediate charge transfer through oxygen vacancies or surface hydroxyls. The results are consistent with previous work [4,5], emphasizing the catalytic importance of oxidized Pt species, particularly Pt^2+^, in redox reactions. The increased activity of SP1a suggests that the combination of high surface hydroxylation, a defect-rich structure, and molecularly dispersed Pt species creates an ideal environment for efficient catalysis. While the SP1c catalyst is the slowest of the three catalysts, it exhibits exceptional structural stability and robustness, making it well suited for operation under extreme conditions. The improved crystallinity of the SnO_2_ support combined with the thermal removal of the hydroxyl groups on the surface and the improved lattice ordering contribute to its resistance to structural degradation at high temperatures. In addition, the strong metal–support interaction between oxidized Pt species (Pt^2+^/Pt^4+^) and the crystalline SnO_2_ support improves the chemical resistance of the catalyst, allowing it to maintain its catalytic activity even in harsh pH environments and under high-pressure conditions [36]. These properties make SP1a promising candidate for catalytic processes where longevity and performance stability are required in demanding industrial or environmental environments [37].

In summary, the combination of surface-sensitive and bulk characterization techniques shows a consistent picture: highly dispersed oxidized Pt species anchored on defect-rich SnO_2_ supports—especially those synthesized under mild conditions—exhibit strong catalytic activity for the reduction of nitroaromatics. The role of synthesis temperature, support purity (almost chloride-free), and surface hydroxyl content is critical in tuning both structural integrity and catalytic performance. This work not only demonstrates the viability of PtOx species as active sites but also provides a scalable and environmentally friendly synthetic strategy for the development of reusable metal oxide catalysts. These findings offer a promising route to the development of cost-effective and environmentally friendly noble metal catalysts for the reduction of nitroaromatic pollutants.

## 5. Conclusions

This study demonstrates that the anion exchange-assisted synthesis route enables the formation of highly dispersed, nanocrystalline SnO_2_ (~5 nm) with a cassiterite structure at near-room temperature, which serves as an efficient support for platinum-based catalysts. Crucially, the anion exchange process not only removes the residual chloride ions from the SnCl_4_ precursors but also produces SnO_2_ nanocrystals with excellent short-term dispersion stability in aqueous media without the need for additional stabilizers. The chloride-free SnO_2_ support enabled a strong interaction between H_2_PtCl_6_ and the SnO_2_ surface, allowing the deposition of platinum at near-room temperature—well below the conventional thermal treatment of ~300 °C normally required for the decomposition of Pt precursors. As a result, the Pt species were effectively dispersed as oxidized PtOx domains, with only a small proportion of Pt^0^. Despite this, the catalytic activity remained excellent, demonstrating that high dispersion and accessibility of active sites can outweigh the need for fully metallic Pt^0^. Our central hypothesis that the properties of the support are as crucial as those of the active catalytic species was confirmed. Of the three studied supports, SnA (used in SP1a) provided superior catalytic performance despite its lower crystallinity, as it had a smaller particle size, larger surface area and better dispersion, which together provided better accessibility to the active sites. In contrast, supports SnB and SnC with larger and more crystalline particles exhibited poorer catalytic activity and required polyvinylpyrrolidone (PVP) for the stability of the suspension during UV–Vis monitoring of the catalytic reaction, which limited their practical applicability. These findings underscore the importance of adjusting not only the catalytic composition but also the properties of the support, such as surface area, pore accessibility and dispersibility in water. Overall, this chloride-free low-temperature synthesis strategy provides a general and scalable platform for the development of noble metal oxide nanocatalysts with high catalytic efficiency and operational robustness.

## Figures and Tables

**Table 1:** The values of volume-averaged domain size (*D*_v_) and upper limits of microstrains (*e*) of the cassiterite phase in the samples SP1a, SP1b, and SP1c, as determined from the results of Rietveld refinements (MAUD program v.2.9995) of samples.

Sample	Phase	Rietveld Refinement		
		*D*_v_/nm	*e* × 10^3^	*R*_wp_
SP1a	Cassiterite	5.0(1)	5.0(1)	0.056
SP1b	Cassiterite	5.1(1)	4.9(1)	0.061
SP1c	Cassiterite	13(1)	1.4(1)	0.053

**Table 2:** Average oxidation state (AOS) of tin (Sn) and platinum (Pt) determined from the XPS results ^i^.

Sample	AOS of Sn	AOS of Pt
SP1a	3.40	1.70
SP1b	3.58	2.14
SP1c	3.35	2.00
SP10a	3.30	1.74

^i^ The average XPS oxidation state of Sn is calculated using the following equation: Sn (average oxidation state) = mole fraction Sn(IV) × 4 + mole fraction Sn(II) × 2 + mole fraction Sn(0) × 0, where the mole fraction corresponds to the XPS %/100 and these values are given above in Table 2. The same model is used for the average oxidation state of Pt.

**Table 3:** Room-temperature ^119^Sn Mössbauer parameter values ^i^ obtained via the fit of the spectra shown in Figure 12.

Sample	$\chi_{n}^{2}$	*δ*, mm s^−1^	Δ, mm s^−1^	*W*_L_, mm s^−1^
SP1a	1.11	0.023(2)	0.544(8)	1.24(1)
SP1b	0.93	0.021(2)	0.529(8)	1.12(1)
SP1c	1.01	0.014(1)	0.523(5)	1.041(6)

^i^ $\chi_{n}^{2}$ denotes the normalized chi-square of the fit, *δ* is the ^119^Sn isomer shift with respect to that of our SnO_2_ standard, Δ is the quadrupole splitting, and *W*_L_ is the Lorentzian FWHM width of the individual lines of the doublet. Numbers in brackets denote the standard fit error (1σ) in the last digit(s).

## Data Availability

The original contributions presented in this study are included in the article/Supplementary Materials. Further inquiries can be directed to the corresponding author(s).

## Supplementary Materials

The following supporting information can be downloaded at: https://www.mdpi.com/article/10.3390/nano15151159/s1, Table S1: Synthesis condition summary for supports SnA, SnB, and SnC and samples SP1a, SP1b, SP1c, and SP10a; Figure S1: XRD patterns of supports SnA, SnB, and SnC and samples SP1a, SP1b, and SP1c; Figure S2: STEM micrograph of sample SP10a, with the diameter of platinum particles depicted with yellow lines; Figure S3: STEM image of sample SP10a (a) and corresponding EDXS elemental mapping images of Sn L edge (b), Pt M edge (c), O K edge (d), and superposition of Sn L, Pt M, and O K edges (e). The EDXS spectrum in (f) confirms the presence of platinum and contains a small amount of chloride, as shown in the table; Figure S4: Thermogravimetric analysis (TGA—black curve), derivative thermogravimetry (DTG—blue curve), and differential scanning calorimetry (DSC—red curve) thermograms for sample SP10a, recorded under a nitrogen atmosphere up to 1000 °C; Table S2: The peak positions and relative proportion (%) of Pt^4+^, Pt^2+^, Pt^0^, Sn^4+^, Sn^2+^, and Sn^0^ in the synthesized samples SP1a, SP1b, and SP1c, based on the deconvoluted Pt 4f and Sn 3d spectra; Figure S5: XPS spectra of sample SP10a, measured around Sn 3d (left panel), Pt 4f (middle panel), and O1s (right panel) core levels; Figure S6: Overview XPS spectrum of the SP1a sample showing the elemental composition and the chemical surface states. The amount of Cl is estimated to ~2 mol%; Figure S7: Overview XPS spectrum of the SP10a sample showing the elemental composition and the chemical surface states. The amount of Cl is estimated to ~10 mol%; Figure S8: Raman spectra of SnO_2_ nanoparticles (sample SnA) and SnO_2_ nanoparticles decorated with platinum (samples SP1a and SP10a); Figure S9: Correlation between the obtained values of the ^119^Sn Mössbauer quadrupole splitting and the *W*_L_ Lorentzian line width parameter in the SP1a, SP1b, and SP1c samples; Figure S10: Relative absorption strength—defined as the Mössbauer spectral area normalized to the baseline and the applied sample mass, and expressed relative to sample SP1c—for samples SP1a, SP1b, and SP1c, as calculated from the Mössbauer spectra shown in Figure 12; Figure S11: UV–Vis spectra of the aqueous solution of pure 4-nitrophenol and 4-nitrophenolate ions after the addition of NaBH_4_; Figure S12: UV–Vis spectra of the 1% PVP solution; Figure S13: Catalytic reduction of 4-nitrophenol (4-NP) to 4-aminophenol (4-AP) as a function of time using support SnA containing no platinum; Figure S14: Catalytic reduction of 4-nitrophenol (4-NP) to 4-aminophenol (4-AP) as a function of time using sample SP1a, without the addition of NaBH4. This sample without the addition of NaBH_4_ in aqueous solution with 4-NP was completely inactive for the reduction of 4-NP to 4-AP.

## References

[1] Wacławek S., Padil V.V., Černík M. (2018). Major Advances and Challenges in Heterogeneous Catalysis for Environmental Applications: A Review. Ecol. Chem. Eng. S.

[2] Chong Y., Chen T., Li Y., Lin J., Huang W.-H., Chen C.-L., Jin X., Fu M., Zhao Y., Chen G. (2023). Quenching-Induced Defect-Rich Platinum/Metal Oxide Catalysts Promote Catalytic Oxidation. Environ. Sci. Technol..

[3] Lagunova V., Filatov E., Plyusnin P., Kostin G., Urlukov A., Potemkin D., Korenev S. (2022). Metal-oxide catalysts for CO TOX and PROX processes in the Pt–Cr/Mo/W systems. Int. J. Hydrogen Energy.

[4] Okumura K., Aikawa S., Aoki Y., Abdullahi A., Mohammed M. (2025). Rational Design of Pt Supported Catalysts for Hydrosilylation: Influence of Support and Calcination Temperature. Innov. Chem. Mater. Sustain..

[5] Mukri B.D., Waghmare U.V., Hegde M.S. (2013). Platinum Ion-Doped TiO_2_: High Catalytic Activity of Pt^2+^ with Oxide Ion Vacancy in Ti^4+^_1–*x*_Pt^2+^*_x_*O_2–*x*_ Compared to Pt^4+^ without Oxide Ion Vacancy in Ti^4+^_1–*x*_Pt^4+^*_x_*O_2_. Chem. Mater..

[6] Degler D., de Carvalho H.W.P., Kvashnina K., Grunwaldt J.-D., Weimar U., Barsan N. (2016). Structure and chemistry of surface-doped Pt:SnO_2_ gas sensing materials. RSC Adv..

[7] Lai J., Luque R., Xu G. (2015). Recent Advances in the Synthesis and Electrocatalytic Applications of Platinum-Based Bimetallic Alloy Nanostructures. ChemCatChem.

[8] Xu H., Shang H., Wang C., Du Y. (2020). Ultrafine Pt-Based Nanowires for Advanced Catalysis. Adv. Funct. Mater..

[9] Ramli Z.A.C., Kamarudin S.K. (2018). Platinum-Based Catalysts on Various Carbon Supports and Conducting Polymers for Direct Methanol Fuel Cell Applications: A Review. Nanoscale Res. Lett..

[10] Zhan F., Huang L., Luo Y., Chen M., Tan R., Liu X., Liu G., Feng Z. (2025). Recent advances on support materials for enhanced Pt-based catalysts: Applications in oxygen reduction reactions for electrochemical energy storage. J. Mater. Sci..

[11] Yang Y., Wang Y., Yin S. (2017). Oxygen vacancies confined in SnO_2_ nanoparticles for desirable electronic structure and enhanced visible light photocatalytic activity. Appl. Surf. Sci..

[12] Li K., Wang Q., Zhao Q., Yu H., Yin H. (2025). Enhanced Catalytic Reduction of 4-Nitrophenol over Porous Silica Nanospheres Encapsulating Pt-Sn_x_O_y_ Hybrid Nanoparticles. Catalysts.

[13] Ðurasović I., Štefanić G., Dražić G., Peter R., Klencsár Z., Marciuš M., Jurkin T., Ivanda M., Stichleutner S., Gotić M. (2023). Microwave-Assisted Synthesis of Pt/SnO_2_ for the Catalytic Reduction of 4-Nitrophenol to 4-Aminophenol. Nanomaterials.

[14] Briskeby S.T., Tsypkin M., Tunold R., Sunde S. (2014). Stability of carbon nanofibre-supported platinum catalysts in the presence of chloride under controlled mass-transfer conditions. J. Power Sources.

[15] Gao R., Zhang M., Liu Y., Xie S., Deng J., Ke X., Jing L., Hou Z., Zhang X., Liu F. (2022). Engineering Platinum Catalysts via a Site-Isolation Strategy with Enhanced Chlorine Resistance for the Elimination of Multicomponent VOCs. Environ. Sci. Technol..

[16] Paiva T., Hashimoto T., Carrilho Planca M.J., Thompson G.E. The Effect of Chloride as Catalyst Layer Contaminant on the Degradation of PEMFCs. Proceedings of the IV Iberian Symposium on Hydrogen, Fuel Cells and Advanced Batteries.

[17] Macera L., Daniele V., Mondelli C., Capron M., Taglieri G. (2021). New Sustainable, Scalable and One-Step Synthesis of Iron Oxide Nanoparticles by Ion Exchange Process. Nanomaterials.

[18] Hesse R., Chassé T., Szargan R. (1999). Peak shape analysis of core level photoelectron spectra using UNIFIT for WINDOWS. Anal. Bioanal. Chem..

[19] Marić I., Gotić M., Pustak A., Dražić G., Grenèche J.-M., Jurkin T. (2022). Magnetic δ-FeOOH/Au nanostructures synthesized using γ-irradiation method and their catalytic activity for the reduction of 4-nitrophenol. Appl. Surf. Sci..

[20] Nancollas G.H., Reddy M.M., Tsai F. (1972). An autoclave for the study of crystal growth and dissolution in aqueous solution at high temperature. J. Phys. E Sci. Instrum..

[21] Matsushima Y., Kakinuma N., Takahashi H., Kondo A., Maeda K., Suzuki T., Kambe S. (2017). Thermogravimetric investigation of oxygen deficiency in SnO_2_ reduced at 500 °C or below. Solid State Ion..

[22] Sing K.S.W. (1985). Reporting physisorption data for gas/solid systems with special reference to the determination of surface area and porosity (Recommendations 1984). Pure Appl. Chem..

[23] Tan Y.H., Davis J.A., Fujikawa K., Ganesh N.V., Demchenko A.V., Stine K.J. (2012). Surface area and pore size characteristics of nanoporous gold subjected to thermal, mechanical, or surface modification studied using gas adsorption isotherms, cyclic voltammetry, thermogravimetric analysis, and scanning electron microscopy. J. Mater. Chem..

[24] Zhang Z., Wang Y., Guo T., Hu P. (2025). The Influence of Defect Engineering on the Electronic Structure of Active Centers on the Catalyst Surface. Catalysts.

[25] Zhou J., Zhang M., Hong J., Fang J., Yin Z. (2005). Structural and spectral properties of SnO2 nanocrystal prepared by microemulsion technique. Appl. Phys. A.

[26] Socrates G. (2004). Infrared and Raman Characteristic Group Frequencies: Tables and Charts.

[27] Indris S., Scheuermann M., Becker S.M., Šepelák V., Kruk R., Suffner J., Gyger F., Feldmann C., Ulrich A.S., Hahn H. (2011). Local Structural Disorder and Relaxation in SnO_2_ Nanostructures Studied by ^119^Sn MAS NMR and ^119^Sn Mössbauer Spectroscopy. J. Phys. Chem. C.

[28] Mejía Y.R., Bogireddy N.K.R. (2022). Reduction of 4-nitrophenol using green-fabricated metal nanoparticles. RSC Adv..

[29] Rusdin A., Gazzali A.M., Thomas N.A., Megantara S., Aulifa D.L., Budiman A., Muchtaridi M. (2024). Advancing Drug Delivery Paradigms: Polyvinyl Pyrolidone (PVP)-Based Amorphous Solid Dispersion for Enhanced Physicochemical Properties and Therapeutic Efficacy. Polymers.

[30] Xu C., Qiu Y., Yang X., Gao Z., Wang Z., Liu C., Sun Y., Ma J., Liu L. (2024). High-Performance Catalytic Reduction of 4-Nitrophenol to 4-Aminophenol over Pt Nanoparticles Supported on Co-Al LDH Nanosheets. Crystals.

[31] Ahmed A., Devi G., Kapahi A., Kundan S., Katoch S., Bajju G.D. (2019). Covalently linked porphyrin-graphene oxide nanocomposite: Synthesis, characterization and catalytic activity. J. Mater. Sci. Mater. Electron..

[32] Marić I., Dražić G., Radin E., Peter R., Škrabić M., Jurkin T., Pustak A., Baran N., Mikac L., Ivanda M. (2022). Impact of platinum loading and dispersion on the catalytic activity of Pt/SnO_2_ and Pt/α-Fe_2_O_3_. Appl. Surf. Sci..

[33] Marić I., Šoltić M., Dražić G., van Spronsen M.A., Štefanić G., Ivanda M., Held G., Jurkin T., Bohinc K., Gotić M. (2023). Synthesis of Pt decorated manganese oxide (MnO_2_/Mn_5_O_8_) nanorods and their catalytic activity for the reduction of 4-nitrophenol to 4-aminophenol. Appl. Surf. Sci..

[34] Zhang J.R., Gao L. (2003). Hydrothermal synthesis and characterization of SnO_2_ nanoparticles. Acta Chim. Sin..

[35] Das T.R.D.T.R., Meena M.M.M., Potheher I.V.P.I.V., Udhaya P.A.U.P.A. (2020). Hydrothermal Synthesis and Characterization of Tin Oxide (SnO_2_) Nanoparticles. J. Environ. Nanotechnol..

[36] Yang G., Lee C. (2022). Building strong metal-support interactions into carbon-supported catalysts for fuel cells. Chem. Catal..

[37] Isahak W.N.R.W., Al-Amiery A. (2024). Catalysts driving efficiency and innovation in thermal reactions: A comprehensive review. Green Technol. Sustain..

