# Supplementary material for: Catalytically Active Oxidized PtOx Species on SnO2 Supports Synthesized via Anion Exchange Reaction for 4-Nitrophenol Reduction
Source: Nanomaterials (Basel). 2025 Jul 28;15(15):1159. doi: 10.3390/nano15151159 (PMC12348751; doi:10.3390/nano15151159)
Supplement: Supplementary file 1 [file nanomaterials-15-01159-s001.zip › nanomaterials-3770505-supplementary.pdf]

Supplementary materials

Catalytically Active Oxidized PtOx Species on SnO_2 Supports Synthesized via Anion Exchange Reaction for 4-Nitrophenol Reduction

Izabela Ðurasovic´ [1], Robert Peter [2], Goran Dražic´ [3], Fabio Faraguna [4], Rafael Anelic´ [4], Marijan Marciuš [5], Tanja Jurkin [6], Vlasta Mohaˇcek Grošev [1], Maria Gracheva [7], Zoltán Klencsár [7], Mile Ivanda [1], Goran Štefanic´ [1,*] and Marijan Gotic´ [1,*]

[1] Laboratory for Molecular Physics and Synthesis of New Materials, Division of Materials Physics, Ru ¯der Boškovic´ Institute, Bijeniˇcka c. 54, 10000 Zagreb, Croatia; idjuras@irb.hr (I.Ð.); mohacek@irb.hr (V.M.G.); ivanda@irb.hr (M.I.)
[2] Department of Physics, University of Rijeka, Radmile Matejˇcic´ 2, 51000 Rijeka, Croatia; rpeter@phy.uniri.hr
[3] National Institute of Chemistry, Hajdrihova 19, SI-1001 Ljubljana, Slovenia; goran.drazic@ki.si
[4] Petroleum and Petrochemical Department, Faculty of Chemical Engineering and Technology, University of Zagreb, Trg Marka Marulic´a Trg 19, 10000 Zagreb, Croatia; ffaragun@fkit.unizg.hr (F.F.); ranelic@fkit.unizg.hr (R.A.)
[5] Division of Materials Chemistry, Ru ¯der Boškovic´ Institute, Bijeniˇcka c. 54, 10000 Zagreb, Croatia; marijan.marcius@irb.hr
[6] Radiation Chemistry and Dosimetry Laboratory, Division of Materials Chemistry, Ru ¯der Boškovic´ Institute, Bijeniˇcka c. 54, 10000 Zagreb, Croatia; tjurkin@irb.hr
[7] Budapest Neutron Centre, HUN-REN Centre for Energy Research, Konkoly-Thege Miklós út 29-33, 1121 Budapest, Hungary; maria.gracheva@ek.hun-ren.hu (M.G.); klencsar.zoltan@ek.hun-ren.hu (Z.K.)
* Correspondence: goran.stefanic@irb.hr (G.Š.); gotic@irb.hr (M.G.)

Table of contents:

Table S1 Synthesis condition summary for supports SnA, SnB, SnC and samples SP1a, SP1b, SP1c and SP10a.

Figure S1 XRD patterns of supports SnA, SnB, SnC and samples SP1a, SP1b and SP1c.

Figure S2 STEM micrograph od sample SP10a, with the diameter of platinum particles depicted with yellow lines.

Figure S3 STEM image of sample SP10a (a) and corresponding EDXS elemental mapping images of Sn L edge (b), Pt M edge (c), O K edge (d), superposition of Sn L, Pt M, and O K edges (e). The EDXS spectrum in (f) confirms the presence of platinum and contains a small amount of chloride, shown in the table.

Figure S4 Thermogravimetric Analysis (TGA - black curve), Derivative Thermogravimetry (DTG - blue curve), and Differential Scanning Calorimetry (DSC - red curve) thermograms for sample SP10a, recorded under a nitrogen atmosphere up to 1000 °C.

Table S2 The peak positions and relative proportion (%) of Pt^{4+}, Pt^{2+}, Pt^{0}, Sn^{4+}, Sn^{2+} and Sn^{0} in the synthesized samples SP1a, SP1b and SP1c, based on the deconvoluted Pt 4f and Sn 3d spectra.

Figure S5 XPS spectra of sample SP10a, measured around Sn 3d (left panel), Pt 4f (middle panel), and O1s (right panel) core levels.

Figure S6 Overview XPS spectrum of the SP1a sample showing the elemental composition and the chemical surface states.

Figure S7 Overview XPS spectrum of the SP10a sample showing the elemental composition and the chemical surface states.

Figure S8 Raman spectra of SnO_2 nanoparticles (sample SnA) and SnO_2 nanoparticles decorated with platinum (samples SP1a and SP10a).

Figure S9 Correlation between the obtained values of the ^{119}Sn Mössbauer quadrupole splitting and the W_L Lorentzian line width parameter in the samples SP1a, SP1b and SP1c.

Figure S10 Relative absorption strength—defined as the Mössbauer spectral area normalized to the baseline and the applied sample mass, and expressed relative to sample SP1c—for samples SP1a, SP1b, and SP1c, as calculated from the Mössbauer spectra shown in Figure 11.

Figure S11 UV-Vis spectra of the aqueous solution of pure 4-nitrophenol and 4-nitrophenolate ions after addition of $NaBH_4$.

Figure S12 UV-Vis spectra of the 1% PVP solution.

Figure S13 Catalytic reduction of 4-nitrophenol (4-NP) to 4-aminophenol (4-AP) as a function of time using support SnA containing no platinum.

Figure S14 Catalytic reduction of 4-nitrophenol (4-NP) to 4-aminophenol (4-AP) as a function of time using sample SP1aA, without the addition of NaBH4. This sample is completely inactive for the reduction of 4-NP to 4-AP.

1. Synthesis of the Supports and Samples

Table S1 Synthesis condition summary for supports SnA, SnB, SnC and samples SP1a, SP1b, SP1c and SP10a.

Sample	Anion Exchange	Hydrothermal Treatment	Annealing	Pt loading / mol%
SnA	☑	☒	☒	0
SnB	☑	☑	☒	0
SnC	☑	☑	☑	0
SP1a	☑	☒	☒	1
SP1b	☑	☑	☒	1
SP1c	☑	☑	☑	1
SP10a	☑	☒	☒	10

2. XRD Results

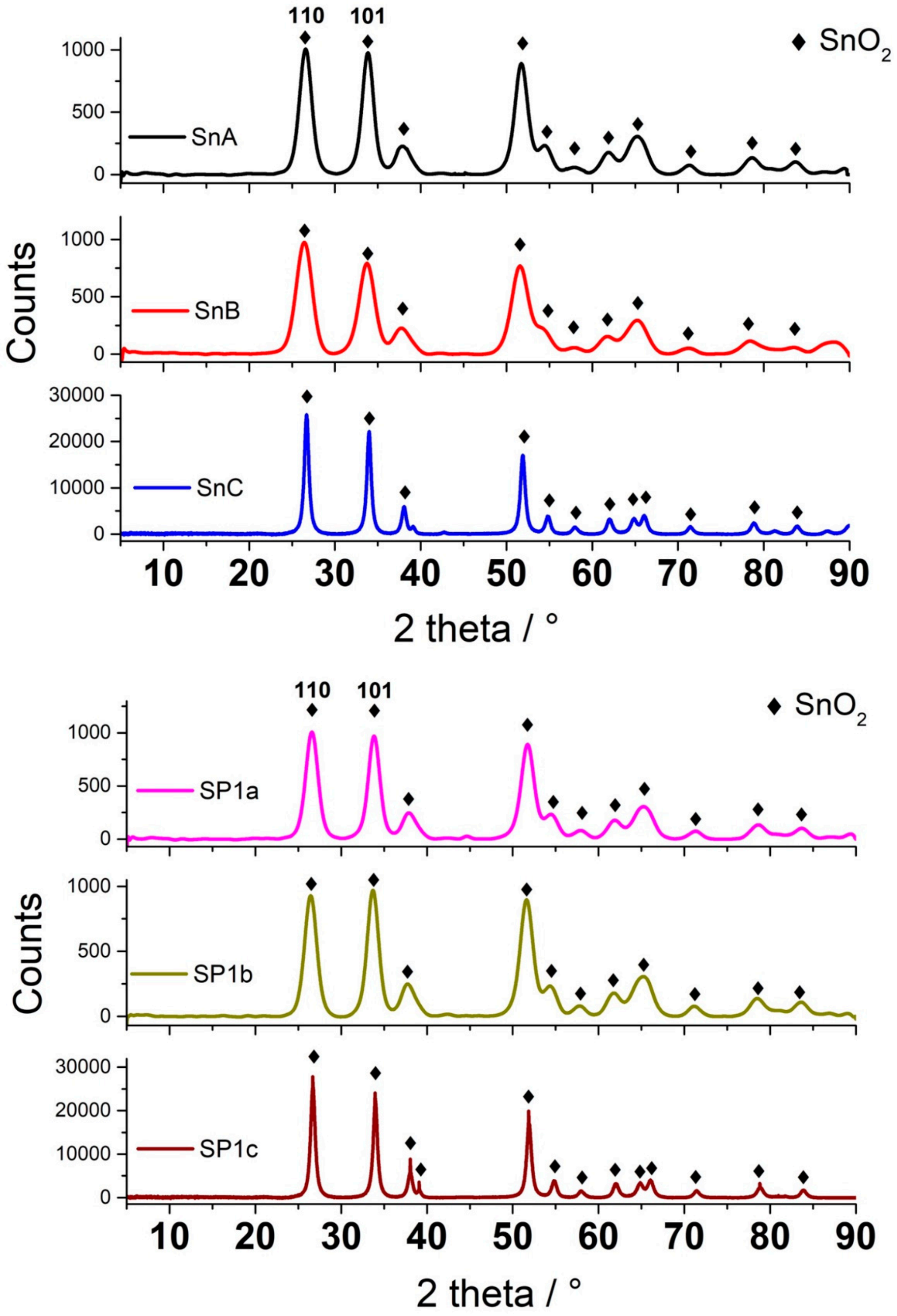

Figure S1 XRD patterns of supports SnA, SnB, SnC and samples SP1a, SP1b and SP1c. SnO_2 = phase structurally closely related to cassiterite (ICDD Card no. 41-1445).

3. STEM Results

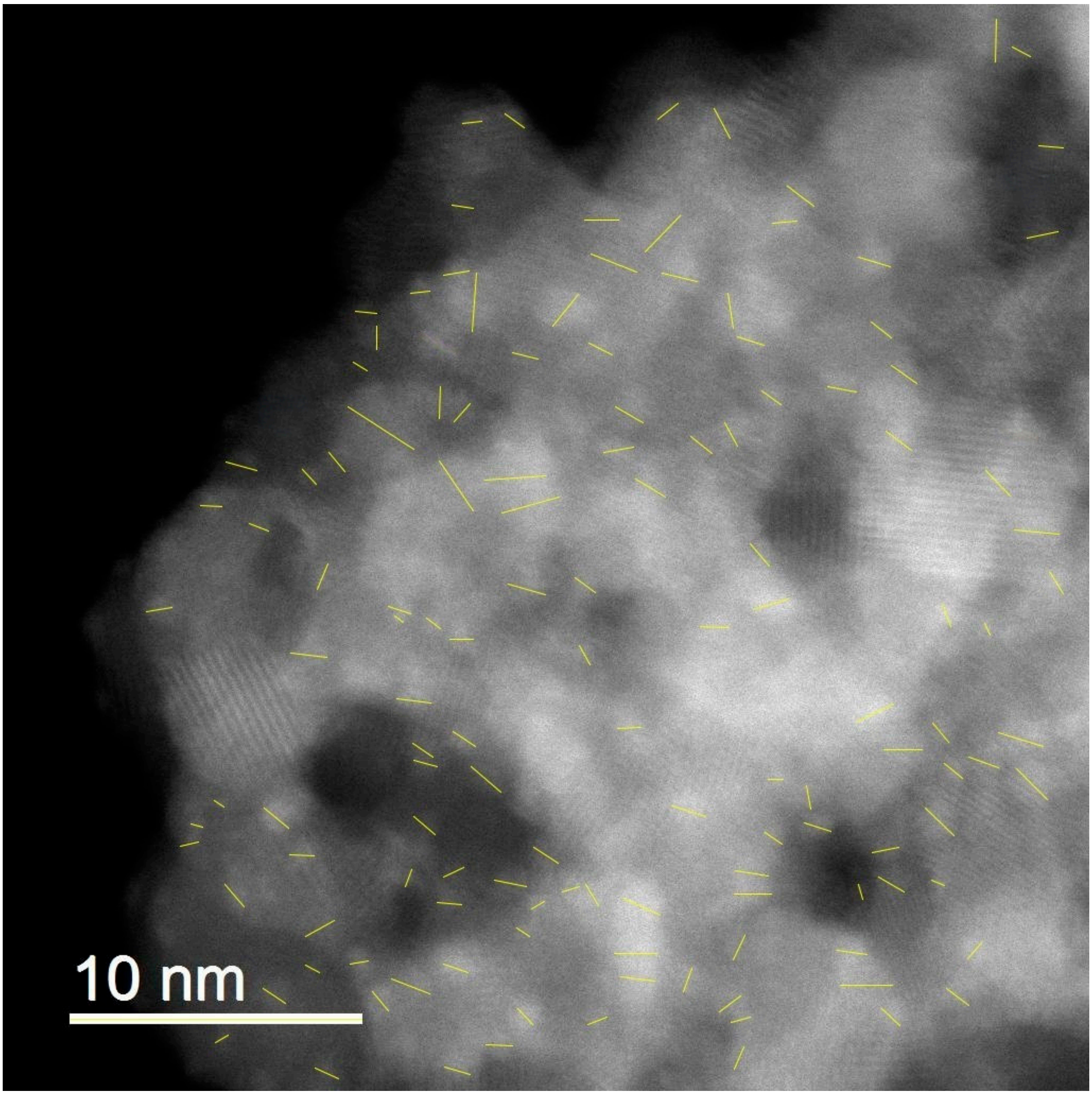

Figure S2 STEM image of the SP10a sample, with yellow line markers (added using the ImageJ software) indicating the measured dimensions of the platinum related (PtOx) nanoparticles.

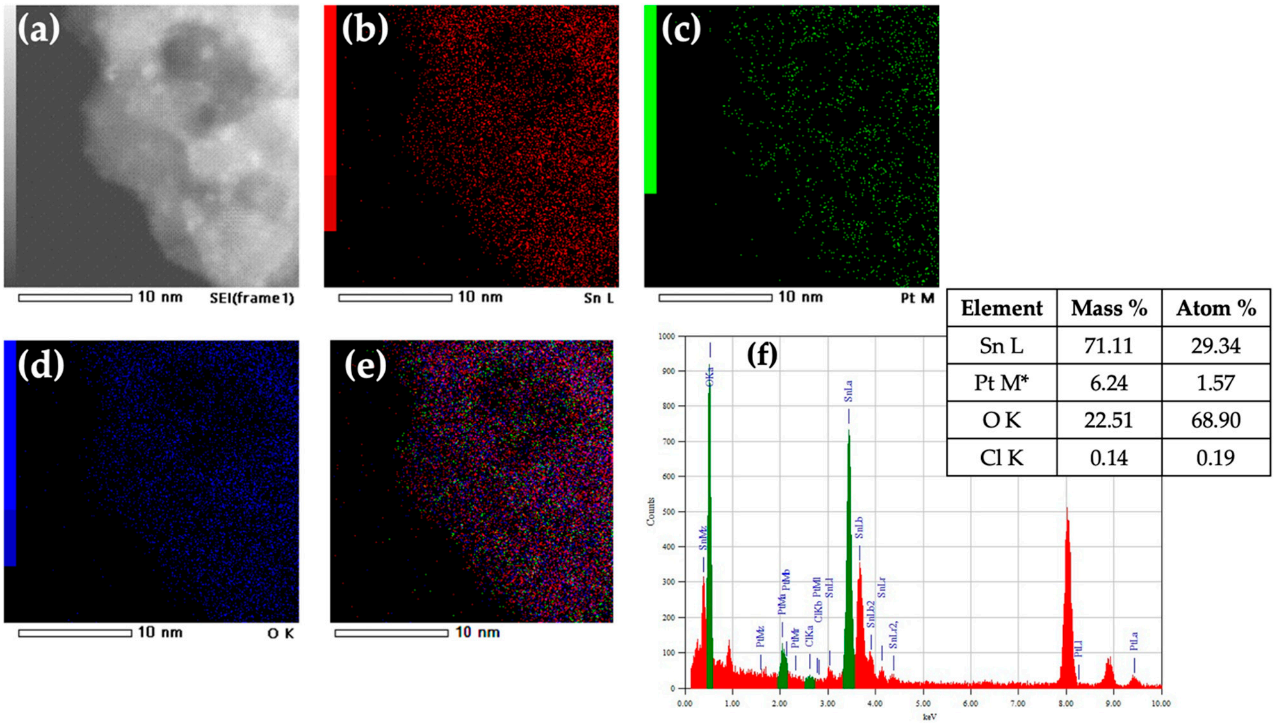

Element	Mass %	Atom %
Sn L	71.11	29.34
Pt M*	6.24	1.57
O K	22.51	68.90
Cl K	0.14	0.19

Figure S3 STEM image of sample SP10a (a) and corresponding EDXS elemental mapping images of Sn L edge (b), Pt M edge (c), O K edge (d), superposition of Sn L, Pt M, and O K edges (e). The EDXS spectrum in (f) confirms the presence of platinum and contains a small amount of chloride, shown in the table.

4. *TGA Analysis*

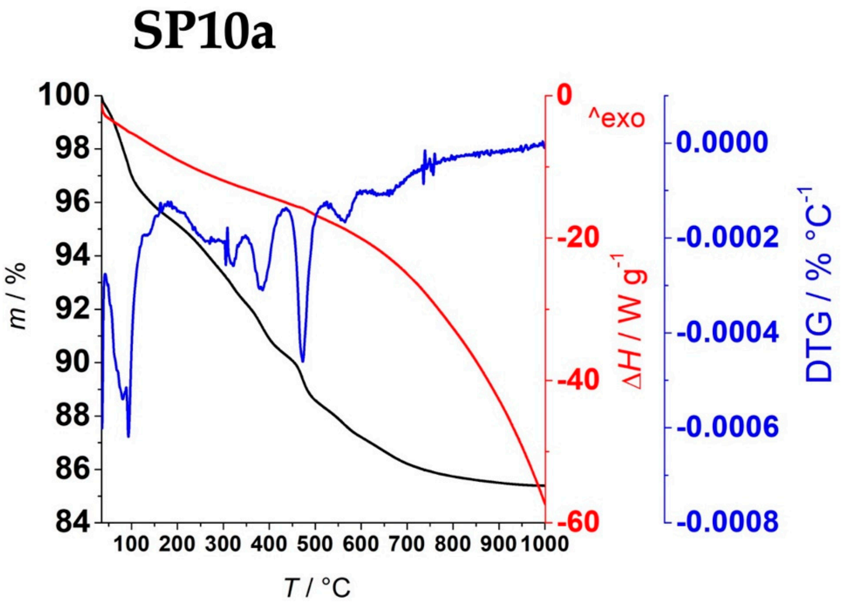

Figure S4 Thermogravimetric Analysis (TGA - black curve), Derivative Thermogravimetry (DTG - blue curve), and Differential Scanning Calorimetry (DSC - red curve) thermograms for sample SP10a, recorded under a nitrogen atmosphere up to 1000 °C.

The TGA curve reveals a total weight loss of approximately 15% across the entire temperature range, indicating the removal of various volatile components. The initial significant weight loss, observed predominantly below ~200 °C with a sharp DTG peak around 80-150 °C and a corresponding broad endothermic event in the DSC, is characteristic of the desorption of physisorbed water molecules and weakly bound chemisorbed water from the highly porous SnO_2 nanoparticles. Following this, a more gradual but continuous weight loss occurs between ~200 °C and ~500 °C, contributing an additional ~5-7% to the total loss. The DTG curve in this region exhibits multiple, less intense peaks (e.g., around 250-300 °C and 350-400 °C), accompanied by subtle exothermic and endothermic features in the DSC. This complex region is likely associated with the decomposition of more strongly bound species, including the dehydroxylation of surface Sn-OH or Pt-OH groups (releasing water), and crucially, the decomposition or desorption of residual chloride species originating from the H_2PtCl_6 precursor. The exothermic events observed might also correspond to the crystallization or reorganization of the amorphous PtO_x domains into more stable oxide phases. Above ~500 °C up to 1000 °C, a very slow, continuous weight loss (approximately 2-3%) persists, attributed to the complete removal of very strongly chemisorbed species or highly stable surface hydroxyl groups. Correlating these thermal events with the Raman spectra of SP10a, the initial weight loss corresponding to water removal is consistent with the general nature of high surface area nanomaterials. The decomposition of chloride species between 200-500 °C strongly supports the presence of Pt-Cl vibrational bands observed in the Raman spectra of SP10a (280-360 cm^{-1}). Furthermore, XPS elemental analysis confirms a substantial increase in chlorine content in SP10a (~10 at.%) compared to SP1a (~2 at.%), whereas STEM-EDX analysis shows no significant Cl signal increase, suggesting that the chlorine detected by XPS is primarily surface-bound. This discrepancy highlights the surface sensitivity of XPS and the

possible inhomogeneous distribution of residual Cl species confined to the outermost layers. The thermal events leading to the crystallization or reorganization of PtO_x (as suggested by DSC exothermic features) could also contribute to the improved crystallinity of the SnO_2 matrix and the formation of distinct Pt-O-Sn interfacial bonds (Raman bands at ~710 cm^{-1}).

Thus, the thermal behaviour of SP10a reflects a hydrated SnO_2 matrix with highly dispersed surface-bound PtOx species and residual chloride, stabilised at low synthesis temperature. The observed weight loss, particularly the gradual profile up to ~800 °C, suggests that Pt species are strongly anchored to the support, consistent with the catalytic stability observed in the main study.

5. XPS Results

Table S2 The peak positions and relative proportions (%) of Pt^{4+}, Pt^{2+}, Pt^{0}, Sn^{4+}, Sn^{2+} and Sn^{0} in the synthesized samples SP1a, SP1b, SP1c, and SP10a, based on the deconvoluted Pt 4f and Sn 3d spectra.

**Sample**	**Electron configuration**	**Binding energy / eV**	**Pt / %**	**Electron configuration**	**Binding energy / eV**	**Sn / %**
**SP1a**	Pt^{4+} $4f_{5/2}$	77.1	Pt^{4+} / 0	Sn^{4+} $3d_{3/2}$	495.2	Sn^{4+} / 77.2
	Pt^{4+} $4f_{7/2}$	73.9		Sn^{4+} $3d_{5/2}$	486.6	
	Pt^{2+} $4f_{5/2}$	75.3	Pt^{2+} / 35.3	Sn^{2+} $3d_{3/2}$	493.8	Sn^{2+} / 15.4
	Pt^{2+} $4f_{7/2}$	72.1		Sn^{2+} $3d_{5/2}$	485.3	
	Pt^{0} $4f_{5/2}$	74.0	Pt^{0} / 64.7	Sn^{0} $3d_{3/2}$	492.6	Sn^{0} / 7.4
	Pt^{0} $4f_{7/2}$	70.7		Sn^{0} $3d_{5/2}$	484.1	
	**Pt 4f**	**eV**	**Pt / %**	**Sn 3d**	**eV**	**Sn / %**
**SP1b**	Pt^{4+} $4f_{5/2}$	77.1	Pt^{4+} / 39.1	Sn^{4+} $3d_{3/2}$	495.1	Sn^{4+} / 85.9
	Pt^{4+} $4f_{7/2}$	73.8		Sn^{4+} $3d_{5/2}$	486.6	
	Pt^{2+} $4f_{5/2}$	75.5	Pt^{2+} / 28.6	Sn^{2+} $3d_{3/2}$	493.9	Sn^{2+} / 7.2
	Pt^{2+} $4f_{7/2}$	72.3		Sn^{2+} $3d_{5/2}$	485.4	
	Pt^{0} $4f_{5/2}$	74.0	Pt^{0} / 32.3	Sn^{0} $3d_{3/2}$	492.6	Sn^{0} / 6.9
	Pt^{0} $4f_{7/2}$	70.7		Sn^{0} $3d_{5/2}$	484.1	
	**Pt 4f**	**eV**	**Pt / %**	**Sn 3d**	**eV**	**Sn / %**
**SP1c**	Pt^{4+} $4f_{5/2}$	77.3	Pt^{4+} / 25.1	Sn^{4+} $3d_{3/2}$	494.2	Sn^{4+} / 76.3
	Pt^{4+} $4f_{7/2}$	74.0		Sn^{4+} $3d_{5/2}$	485.8	
	Pt^{2+} $4f_{5/2}$	75.5	Pt^{2+} / 49.9	Sn^{2+} $3d_{3/2}$	493.1	Sn^{2+} / 14.8
	Pt^{2+} $4f_{7/2}$	72.3		Sn^{2+} $3d_{5/2}$	484.5	
	Pt^{0} $4f_{5/2}$	73.9	Pt^{0} /25.0	Sn^{0} $3d_{3/2}$	491.8	Sn^{0} / 8.9
	Pt^{0} $4f_{7/2}$	70.7		Sn^{0} $3d_{5/2}$	483.2	
	**Pt 4f**	**eV**	**Pt / %**	**Sn 3d**	**eV**	**Sn / %**
**SP10a**	Pt^{4+} $4f_{5/2}$	77.3	Pt^{4+} / 21.6	Sn^{4+} $3d_{3/2}$	494.2	Sn^{4+} / 74.4
	Pt^{4+} $4f_{7/2}$	74.0		Sn^{4+} $3d_{5/2}$	485.8	
	Pt^{2+} $4f_{5/2}$	75.5	Pt^{2+} / 43.0	Sn^{2+} $3d_{3/2}$	493.1	Sn^{2+} / 16.0
	Pt^{2+} $4f_{7/2}$	72.3		Sn^{2+} $3d_{5/2}$	484.5	
	Pt^{0} $4f_{5/2}$	73.9	Pt^{0} /34.5	Sn^{0} $3d_{3/2}$	491.8	Sn^{0} / 9.6
	Pt^{0} $4f_{7/2}$	70.7		Sn^{0} $3d_{5/2}$	483.2	

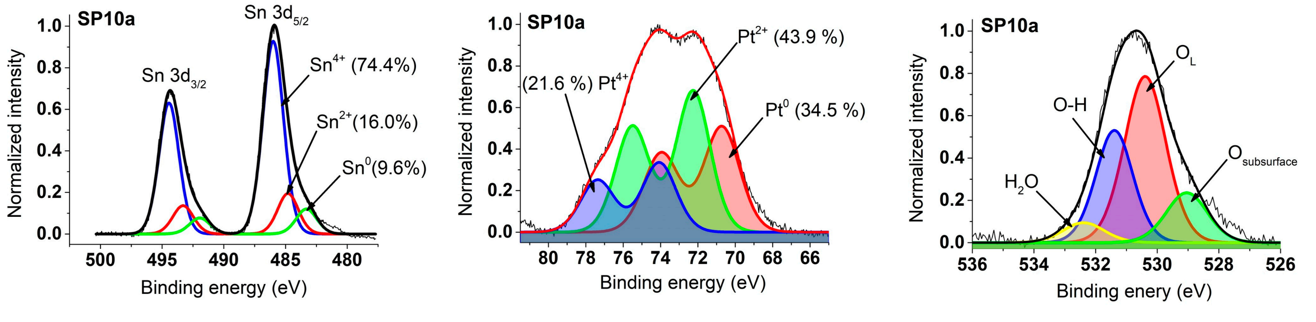

Figure S5 XPS spectra of sample SP10a, measured around Sn 3d (left panel), Pt 4f (middle panel), and O1s (right panel) core levels.

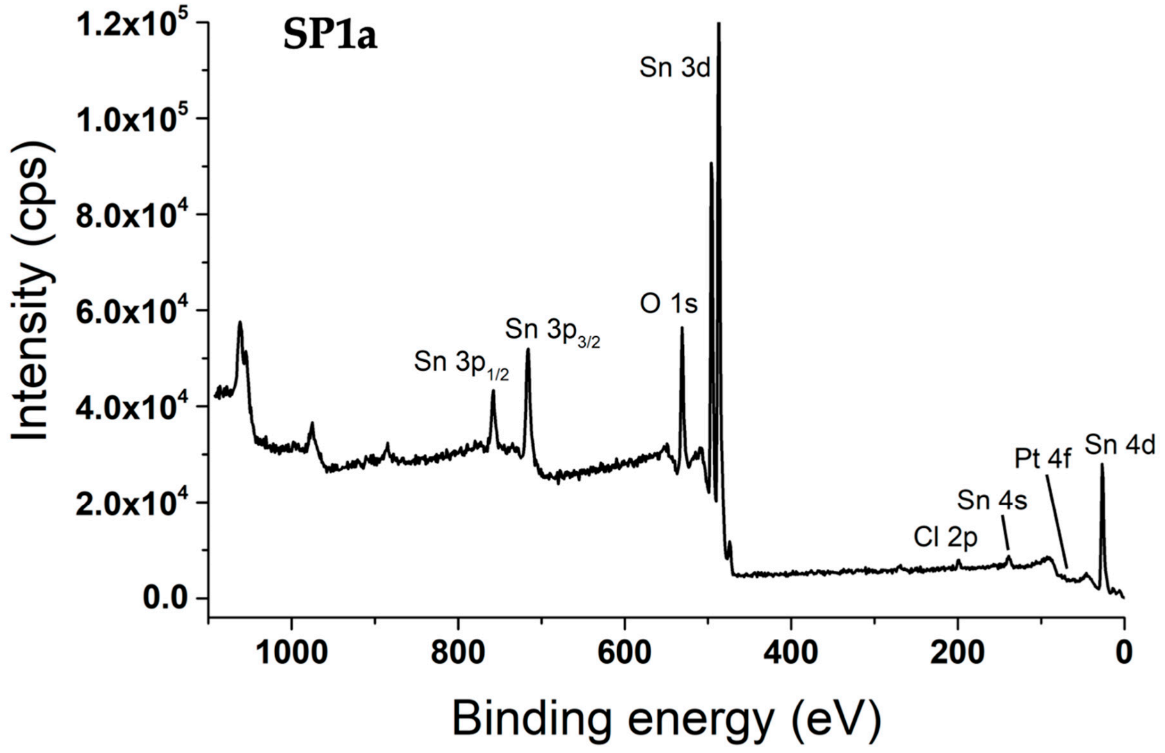

Figure S6 Overview XPS spectrum of the SP1a sample showing the elemental composition and the chemical surface states. The amount of Cl is estimated to ~2 mol%.

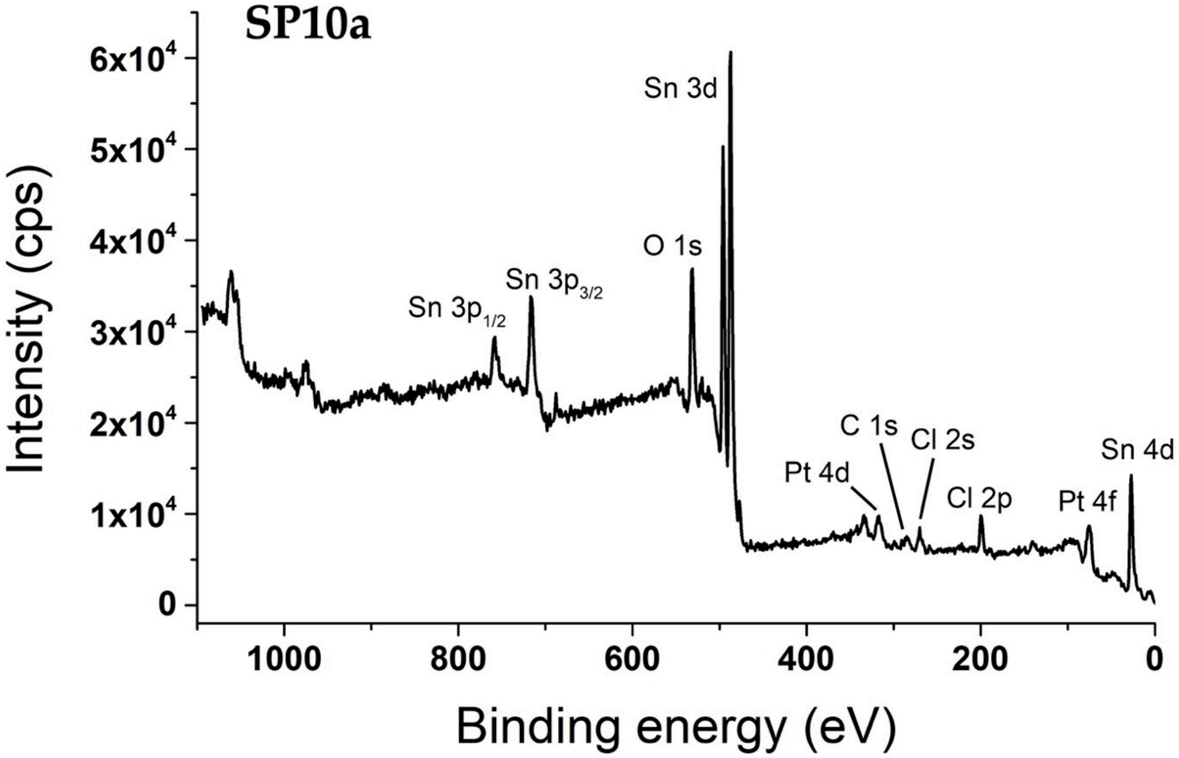

Figure S7 Overview XPS spectrum of the SP10a sample showing the elemental composition and the chemical surface states. The amount of Cl is estimated to ~10 mol%.

Elemental analysis showed that the catalysts do not contain carbon, which is confirmed by the absence of a C 1s signal in the Figure S6 and a small C 1s signal in Figure S7. This indicates that no carbon-based impurities or support materials contribute to the observed catalytic activity, further emphasizing the purity of the SnO_2 support and the exclusive role of the platinum species in driving the catalytic process.

6. Raman Spectroscopy

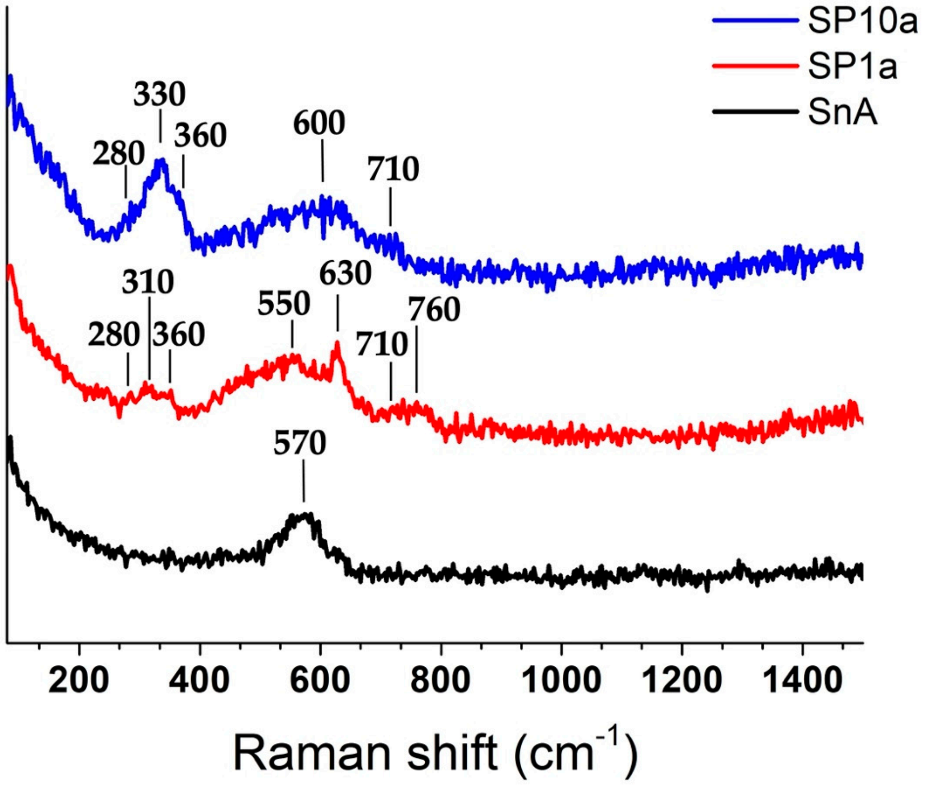

Figure S8 Raman spectra of SnO_2 nanoparticles (sample SnA) and SnO_2 nanoparticles decorated with platinum (samples SP1a and SP10a).

SnA (black curve) serves as a reference for the bare SnO_2 nanoparticles, which have a particle size of ~5 nm, a large surface area of ~130 m^2/g, and a pore size of ~3.2 nm. Samples SP1a and SP10a represent SnO_2 decorated with 1 mol% and 10 mol% Pt, respectively, where Pt is introduced via the hydrolysis of H_2PtCl_6 in an aqueous medium at a low temperature of 37 °C. The platinum is present as 0.85 nm PtO_x domains ("flecks") on the surface, with some contribution from Pt^0 and residual chlorides from the precursor. The Raman spectrum of SnA consist of the most prominent and relatively narrow band at ~570 cm^{-1}, which can be unequivocally assigned to the A_1g vibrational mode of cassiterite SnO_2. While this mode is typically observed around 630 cm^{-1} for bulk SnO_2, a significant downshift and broadening for nanoscale SnO_2 (5 nm) are commonly observed due to phonon confinement effects and contributions from surface vibrational modes stemming from the high surface area (130 m^2/g) and porosity (3.2 nm), confirming the 570 cm^{-1} band as characteristic of the specific SnO_2 nanocrystalline structure. The spectra for SP1a and SP10a show several additional bands and shifts in comparison to the SnA spectrum, which can be attributed to the presence of platinum (in oxide forms as PtOx) and residual chloride species. Bands attributed to Pt/Cl species include those at ~280 cm^{-1}, ~310 cm^{-1}(~330 cm^{-1}) and ~360 cm^{-1}, in samples SP1a and SP10a, which are strongly indicative of Pt-Cl stretching and probably to Pt-O bending vibrations. The precursor H_2PtCl_6 readily hydrolyzes, leading to the formation of various chloroplatinate species, adsorbed chlorides on Pt^0 or PtO_x surfaces, or even chloride-bridged platinum

complexes, with the observed range (280-360 cm^{-1}) consistent with literature values for Pt-Cl vibrations in different platinum coordination environments. The presence of multiple distinct bands suggests a variety of Pt-Cl interactions or different complex geometries on the surface, and the differences in peak positions and intensities between SP1a and SP10a (e.g., the more distinct 330 cm^{-1} band in SP10a) likely reflect variations in the speciation and concentration of these chloride-containing platinum species due to different platinum loadings. The region around ~550 cm^{-1} (in SP1a) and ~600 cm^{-1} (in SP10a) shows a complex interplay between the intrinsic SnO_2 A_1g mode (observed at 570 cm^{-1} in SnA) and a clear additional contribution from Pt-O vibrations within the PtO_x domains (0.85 nm flecks). Platinum oxides, particularly PtO_2, often exhibit Raman bands in the 500-800 cm^{-1} range, with prominent features around 593-600 cm^{-1}, making this overlap highly probable. The broader feature around 550 cm^{-1} in SP1a suggests a more disordered PtO_x phase or a stronger overlap with the SnO_2 band, whereas the sharper, more pronounced shoulder/band around 600 cm^{-1} in SP10a strongly points to the presence of a more crystalline or well-defined PtO_x phase due to the higher platinum loading, which might lead to larger or more ordered PtO_x domains. **The band at ~630 cm^{-1}** in sample SP1a (autoclaved sample), whose appearance and enhanced intensity are notable especially compared to its absence or very weak presence in SnA, suggests the presence of a more crystalline cassiterite SnO_2 phase. This could indicate that the presence of platinum (even at low temperature hydrolysis) and/or the subsequent processing conditions for SP1a and SP10a induce a recrystallization or improved crystallinity of the SnO_2 matrix, as platinum can act as a catalyst for crystal growth, shifting the phonon confinement effect towards a more bulk-like Raman response. Finally, the high-frequency band at ~710 cm^{-1} in SP10a is particularly important for understanding the interfacial interactions and is most likely attributable to bridging Pt-O-Sn vibrations formed at the interface between the PtO_x domains and the SnO_2 nanoparticles, a formation highly plausible given the intimate contact. This band is also present in sample SP1a, though as part of a very broad band at 760 cm^{-1}, effectively making it less distinct. This broader band at 760 cm^{-1} very probably belongs to intrinsic vibrational modes of SnO_2, becoming more evident in SP1a due to its high crystallinity and still good dispersion and large surface area. While other possibilities include enhanced B_2g modes of SnO_2 (typically ~770 cm^{-1}) due to improved crystallinity, or other specific Pt-O species, the appearance of these bands exclusively in the platinum-containing samples strongly points towards Pt-O-Sn interactions. Therefore, the Raman spectra provide valuable insights into the speciation and interactions of platinum on the SnO_2 surface, with the shifts and new bands highlighting the formation of Pt-Cl species, the presence of PtO_x domains (including Pt-O-Pt bonds), and crucially, the likely formation of interfacial Pt-O-Sn bonds, all of which are critical for understanding the catalytic properties of such composite materials.

7. ^{119}Sn Mössbauer Spectroscopy Results

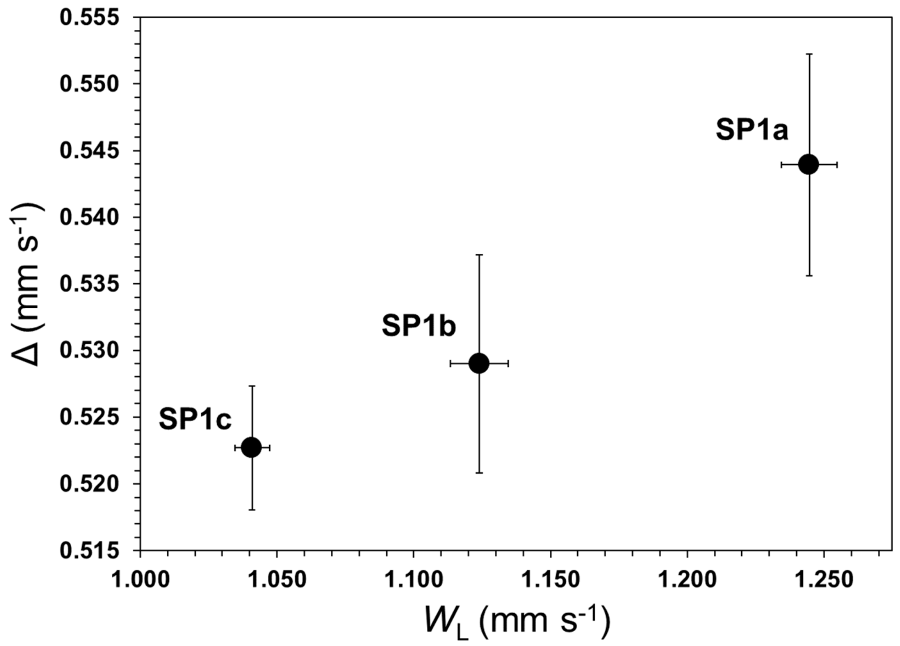

Figure S9 Correlation between the obtained values of the ^{119}Sn Mössbauer quadrupole splitting and the W_L Lorentzian line width parameter in the samples SP1a, SP1b and SP1c.

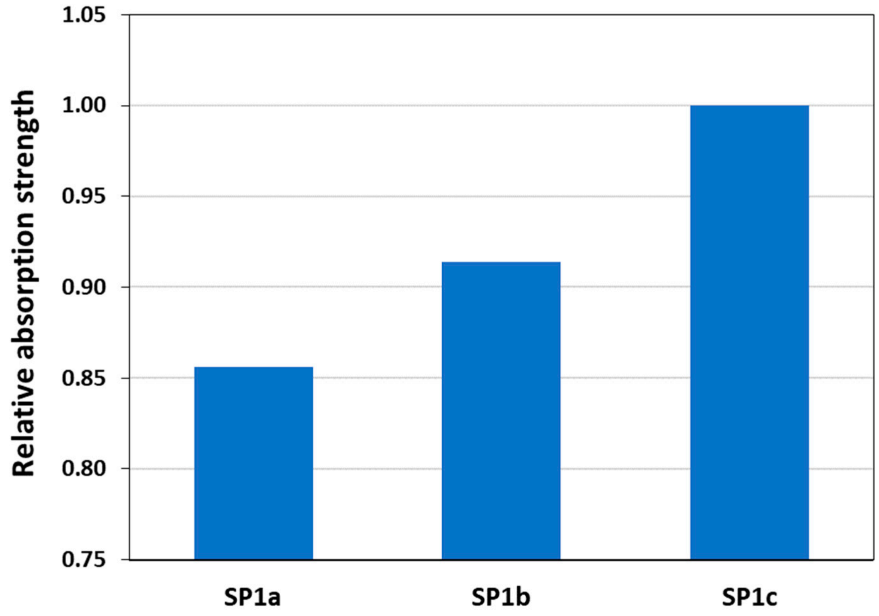

Figure S10 Relative absorption strength—defined as the Mössbauer spectral area normalized to the baseline and the applied sample mass, and expressed relative to sample SP1c—for samples SP1a, SP1b, and SP1c, as calculated from the Mössbauer spectra shown in Figure 12.

8. Catalytic Measurements

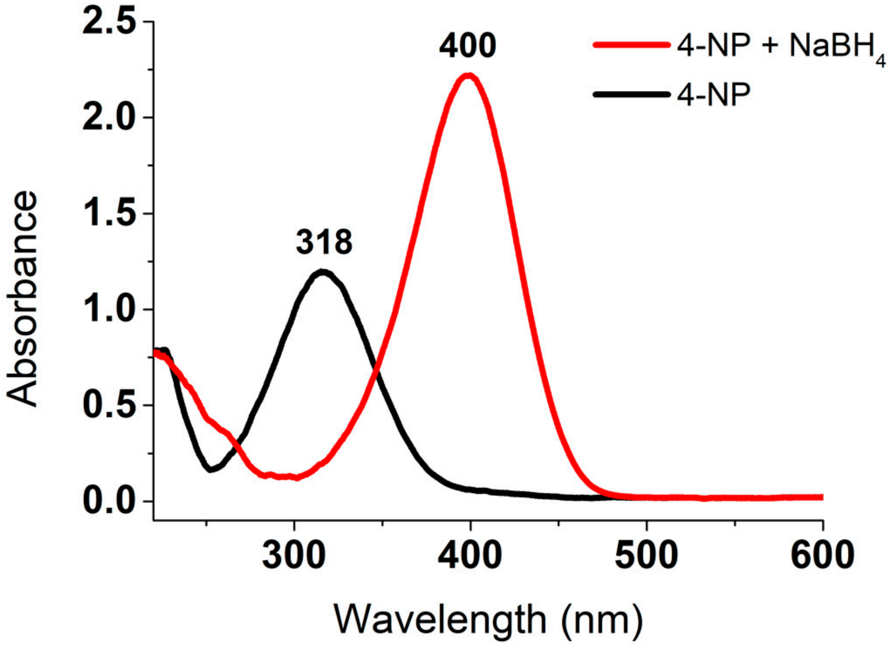

Figure S11 UV-Vis spectra of the aqueous solution of pure 4-nitrophenol with the maximum at 318 nm and 4-nitrophenolate ions after addition of $NaBH_4$ (alkaline aqueous solution) with maximum at 400 nm.

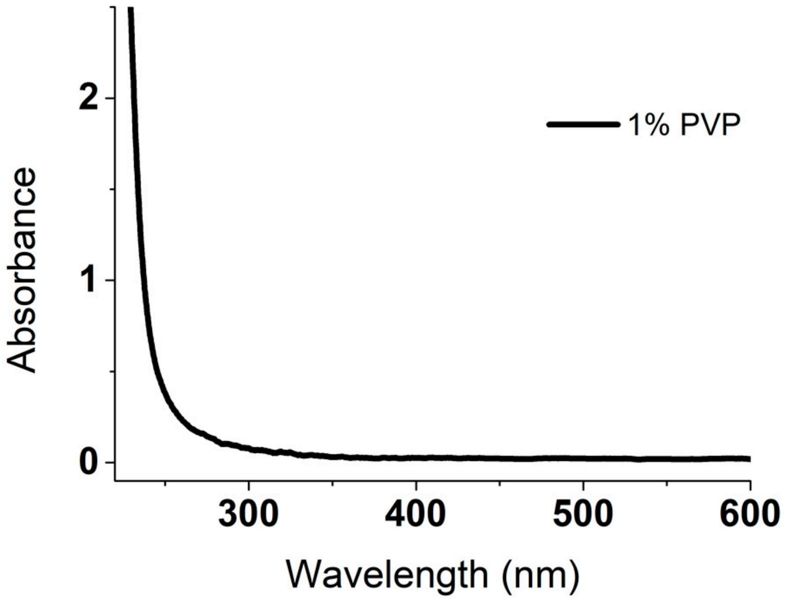

Figure S12 UV-Vis spectra of the 1% PVP solution.

This spectra is recorded to ensure that the polyvinylpyrrolidone (PVP) used as a stabilizing agent does not interfere with the catalytic reduction of 4-nitrophenol to 4-aminophenol. The recorded spectra confirmed that PVP does not absorb in the spectral regions relevant for the monitoring of 4-nitrophenol (400 nm) and 4-aminophenol (300 nm) and also does not generate overlapping bands. This confirms that PVP does not contribute to or interfere with the observed catalytic activity or spectral analysis.

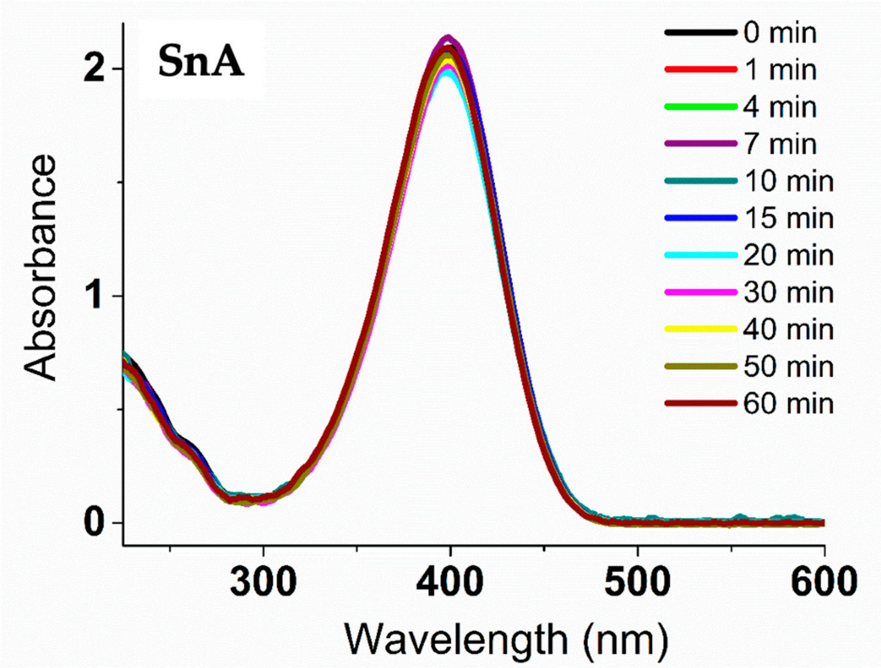

Figure S13 Catalytic reduction of 4-nitrophenol (4-NP) to 4-aminophenol (4-AP) as a function of time using sample SnA, which does not contain platinum. This sample is completely inactive for the reduction of 4-NP to 4-AP.

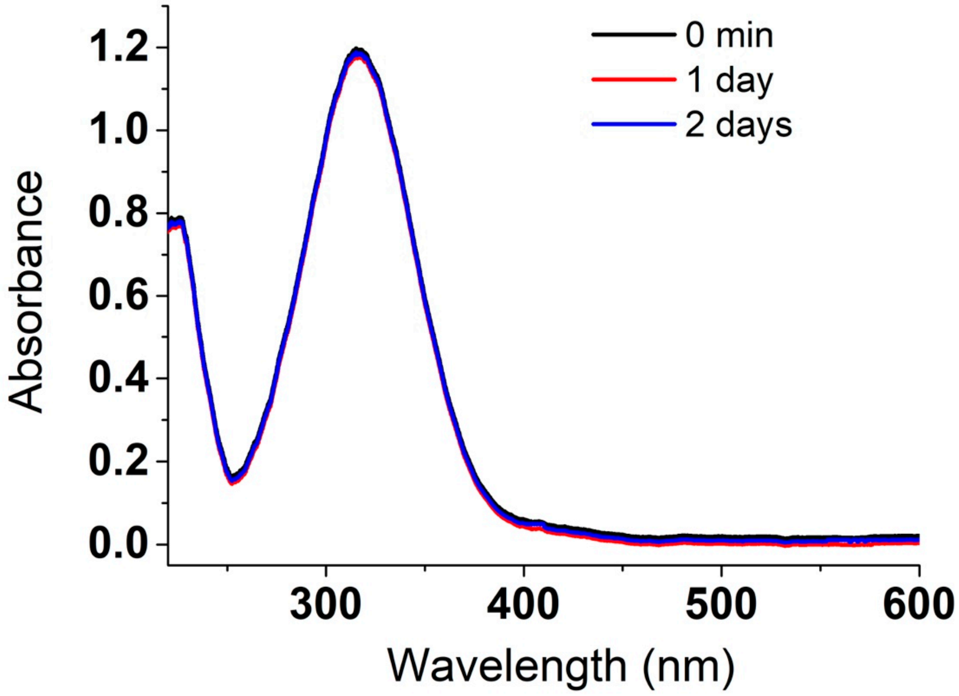

Figure S14 Catalytic reduction of 4-nitrophenol (4-NP) to 4-aminophenol (4-AP) as a function of time using sample SP1a, without the addition of $NaBH_4$. This sample without the addition of $NaBH_4$ in aqueous solution with 4-NP was completely inactive for the reduction of 4-NP to 4-AP.
